# *In Vivo* Structure-Function Analysis and Redox Interactomes of Leishmania tarentolae Erv

**DOI:** 10.1128/Spectrum.00809-21

**Published:** 2021-09-29

**Authors:** Gino L. Turra, Linda Liedgens, Frederik Sommer, Luzia Schneider, David Zimmer, Jordi Vilurbina Perez, Sasa Koncarevic, Michael Schroda, Timo Mühlhaus, Marcel Deponte

**Affiliations:** a Department of Parasitology, Ruprecht-Karls University, Heidelberg, Germany; b Faculty of Chemistry, TU Kaiserslautern, Kaiserslautern, Germany; c Molecular Biotechnology & Systems Biology, TU Kaiserslautern, Kaiserslautern, Germany; d Computational Systems Biology, TU Kaiserslautern, Kaiserslautern, Germany; e Proteome Sciences R&D GmbH & Co. KG, Frankfurt, Germany; Northwestern University

**Keywords:** *Leishmania*/CRISPR-Cas9/SILAC/Erv/Mia40 replacement, CRISPR-Cas9, Erv, *Leishmania*, Mia40, SILAC, mitochondrial protein import

## Abstract

Import and oxidative folding of proteins in the mitochondrial intermembrane space differ among eukaryotic lineages. While opisthokonts such as yeast rely on the receptor and oxidoreductase Mia40 in combination with the Mia40:cytochrome *c* oxidoreductase Erv, kinetoplastid parasites and other Excavata/Discoba lack Mia40 but have a functional Erv homologue. Whether excavate Erv homologues rely on a Mia40 replacement or directly interact with imported protein substrates remains controversial. Here, we used the CRISPR-Cas9 system to generate a set of tagged and untagged homozygous mutants of *LTERV* from the kinetoplastid model parasite Leishmania tarentolae. Modifications of the shuttle cysteine motif of *Lt*Erv were lethal, whereas replacement of clamp residue Cys^17^ or removal of the kinetoplastida-specific second (KISS) domain had no impact on parasite viability under standard growth conditions. However, removal of the KISS domain rendered parasites sensitive to heat stress and led to the accumulation of homodimeric and mixed *Lt*Erv disulfides. We therefore determined and compared the redox interactomes of tagged wild-type *Lt*Erv and *Lt*Erv^ΔKISS^ using stable isotope labeling by amino acids in cell culture (SILAC) and quantitative mass spectrometry. While the Mia40-replacement candidate Mic20 and all but one typical substrate with twin Cx_3/9_C-motifs were absent in both redox interactomes, we identified a small set of alternative potential interaction partners with putative redox-active cysteine residues. In summary, our study reveals parasite-specific intracellular structure-function relationships and redox interactomes of *Lt*Erv with implications for current hypotheses on mitochondrial protein import in nonopisthokonts.

**IMPORTANCE** The discovery of the redox proteins Mia40/CHCHD4 and Erv1/ALR, as well as the elucidation of their relevance for oxidative protein folding in the mitochondrial intermembrane space of yeast and mammals, founded a new research topic in redox biology and mitochondrial protein import. The lack of Mia40/CHCHD4 in protist lineages raises fundamental and controversial questions regarding the conservation and evolution of this essential pathway. Do protist Erv homologues act alone, or do they use the candidate Mic20 or another protein as a Mia40 replacement? Furthermore, we previously showed that Erv homologues in L. tarentolae and the human pathogen L. infantum are not only essential but also differ structurally and mechanistically from yeast and human Erv1/ALR. Here, we analyzed the relevance of such structural differences *in vivo* and determined the first redox interactomes of a nonopisthokont Erv homologue. Our data challenge recent hypotheses on mitochondrial protein import in nonopisthokonts.

## INTRODUCTION

Mitochondrial protein import is an essential process that ensures the biogenesis and diverse functions of mitochondria in eukaryotes (1). Even though eukaryotes use specific, conserved signals for protein targeting to the matrix, the inner membrane, the intermembrane space, or the outer membrane, several key receptors and other components of the well-characterized mitochondrial protein import machineries from opisthokonts are missing or are drastically altered in other major eukaryotic lineages (2–4). One of these lineages, the Excavata/Discoba, include important pathogens such as kinetoplastid parasites of the genera *Leishmania* and *Trypanosoma* (5). The protein import of a major group of proteins in the mitochondrial intermembrane space (IMS) depends on the formation of structural disulfide bonds and oxidative protein folding (6–8). In yeast and other opisthokonts, this process involves (i) Mia40, which acts as a receptor and initial dithiol:disulfide oxidoreductase that recognizes and oxidizes the incoming substrate, and (ii) the flavoprotein Erv1, which reoxidizes Mia40 and transfers two single electrons to cytochrome *c* and the respiratory chain (or to oxygen or fumarate) (9–20). Common substrates of the Mia40/Erv1-import pathway include the small Tims and other essential as well as nonessential proteins with twin Cx_3_C or twin Cx_9_C motifs (9–11, 21). Homologues of several of these substrates are conserved and are also present in apicomplexan and kinetoplastid parasites (although the small Tims in kinetoplastid parasites reflect hybrid proteins that combine different motifs of their opisthokont homologues) (2, 22–24). Furthermore, while Erv homologues are found in all major eukaryotic lineages, Mia40 is absent in many protists, including, for example, apicomplexan and kinetoplastid parasites (2, 22, 25–28). The conservation of Erv and substrate homologues in contrast to the frequent absence of Mia40 homologues in eukaryotes raises controversial questions regarding the evolution of this import pathway and the necessity of a Mia40 replacement that acts as a receptor and/or oxidoreductase (2, 25, 26, 28–32). For example, recent RNA interference (RNAi) analysis of Trypanosoma brucei revealed a decreased import of IMS proteins following the downregulation of the thioredoxin-like protein *Tb*Mic20, a component of the mitochondrial contact site and cristae organization system (MICOS) (31). Although *Tb*Mic20 is an interesting Mia40-replacement candidate, there is no direct evidence for its receptor or oxidoreductase activity to date. In summary, while some studies favor a scenario with Erv as a single-component machinery (29, 30) others point toward a two-component machinery for the import of IMS proteins in Excavata/Discoba (27, 31, 32).

**FIG 1:**
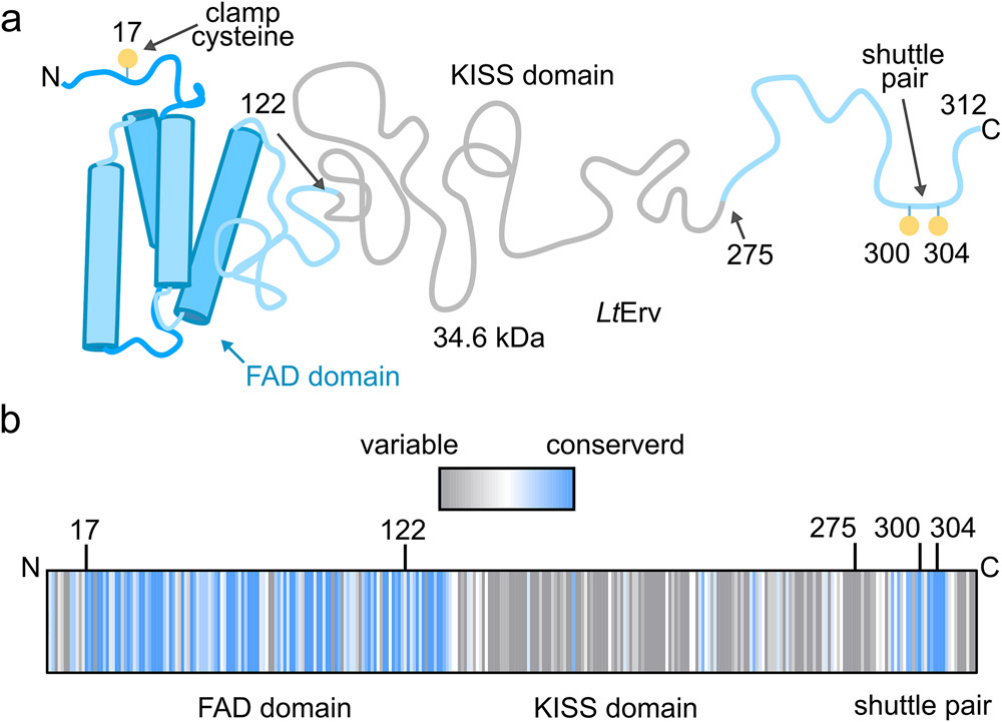
Schematic representation of *Lt*Erv. (a) Residue Cys^17^, the FAD-binding flavodomain with its two active-site and two structural cysteine residues (not shown), and the two shuttle cysteine residues at the C-terminal arm are conserved among Erv homologues from kinetoplastid parasites. The variable KISS domain (residues 122 to 275) between the flavodomain and the shuttle arm is absent in other eukaryotes. (b) The degree of conservation of each residue of *Lt*Erv was calculated based on the sequences of 24 Erv homologues from kinetoplastid parasites using ConSurf (72).

The Erv-encoding genes in Leishmania infantum and Leishmania infantum tarentolae are essential (32, 33), and endogenous and heterologous *Lt*Erv was shown to be imported into the IMS in L. tarentolae and in yeast, respectively (2, 28). Furthermore, RNAi knockdowns of *Tb*Erv1 caused a growth arrest (27), impaired the mitochondrial protein import of 13 proteins with twin Cx_3,9_C motifs, and led to the identification of 25 candidate substrates in the IMS of T. brucei (24). Structurally, Erv homologues from kinetoplastid parasites share (i) a clamp cysteine residue close to the N terminus, (ii) the α-helical Erv/ALR flavodomain with a proximal active site cysteine pair and a structural cysteine pair, (iii) a variable kinetoplastida-specific second (KISS) domain, and (iv) a C-terminal arm with a distal shuttle cysteine pair that can transfer electrons from the protein surface to the active site (Fig. 1) (26, 28, 32). The relevance, function and structure of the KISS domain are unknown. Structure-function analyses in yeast revealed that *Lt*Erv cannot replace *Sc*Erv1 unless the clamp residue Cys^17^ of *Lt*Erv is replaced by serine (28, 32). Furthermore, neither *Lt*Erv nor *Lt*Erv^C17S^ could compensate the loss of *Sc*Mia40 in yeast (32). Similar to yeast *Sc*Erv1 (34) and its human homologue ALR (13), recombinant *Lt*Erv and *Tb*Erv1 were shown to preferentially transfer electrons from dithiothreitol (DTT) to cytochrome *c* (27, 28) and are therefore sulfhydryl:cytochrome *c* electron transferases (EC 1.8.2) and not oxidases (EC 1.8.3) (26, 28). The physiological reducing agents of *Lt*Erv and *Tb*Erv1, which might also include thiols with functions other than mitochondrial protein import, are so far unknown.

We used the recently established CRISPR-Cas9 system in L. tarentolae to perform *Lt*Erv structure-function analyses *in vivo* and to determine its redox interactome. We show that the shuttle cysteine motif of *Lt*Erv is crucial for parasite survival, whereas clamp residue Cys^17^ is dispensable. Removal of the KISS domain influences the redox state of *Lt*Erv and renders parasites sensitive to heat stress but has no effect on the growth rate under standard conditions. Furthermore, the redox interactomes of parasites with tagged wild-type and KISS-less *Lt*Erv revealed neither direct interactions with Mic20 nor canonical substrates but a small set of alternative potential interaction partners with conserved cysteine motifs.

## RESULTS

### Generation and validation of chromosomally tagged *LTERV*.

To purify interaction partners by affinity chromatography and to gain insights into the redox interactome of *Lt*Erv, we generated L. tarentolae cell lines that chromosomally encode *Lt*Erv with a C-terminal His_8_-tag. Using the CRISPR-Cas9 system (33), we introduced a chromosomal double-strand break downstream of the *LTERV* open reading frame and provided a DNA repair fragment with homology regions that flank the tag-coding sequence and a resistance cassette against puromycin (see Fig. S1a in the supplemental material). Two clonal cell lines were characterized following selection on agar plates. Sequencing of a PCR amplicon with chromosomal DNA as a template confirmed the homozygous integration and expected coding sequence for the His_8_ tag (Fig. S1b). Furthermore, PCR analyses confirmed the replacement of wild-type *LTERV* and the correct insertion of the antibiotic resistance cassette (Fig. S1c). The presence of the His tag was also validated for both cell lines by Western blot analysis (Fig. S1d).

The replacement of *Lt*Erv with *Lt*Erv-His_8_ did not affect the growth of L. tarentolae in liquid cultures (Fig. S2a). Furthermore, a pilot experiment confirmed that the bait protein can be purified under nonreducing, denaturing conditions from total cell lysates by affinity chromatography. The protein content of the eluates was analyzed by SDS-PAGE and silver staining. *Lt*Erv was detected by Western blot analyses in these eluate fractions but was absent in negative controls that were purified in parallel from cell lysates of the parental strain (Fig. S2b). In summary, we successfully generated homozygous L. tarentolae cell lines with chromosomally encoded, C-terminally His_8_-tagged *Lt*Erv that can be purified by affinity chromatography.

### Establishment of a SILAC protocol for L. tarentolae.

To perform quantitative mass spectrometry, we established a protocol for SILAC (stable isotope labeling by amino acids in cell culture). Previous attempts to find a suitable medium for L. tarentolae without fetal bovine serum (FBS) (as a confounding source for arginine or lysine) or with dialyzed FBS had failed (33). We therefore tested five alternative medium compositions based on M199, which had been used in SILAC experiments with Leishmania donovani (35) (Fig. S3). Since *Leishmania* spp. are auxotrophic for purines, pterins, and folate (36–38), we tested three compositions (growth conditions 1 to 3) with increased concentrations of hypoxanthine, adenosine, 6-biopterin, and folic acid as supplements in accordance with other defined media for kinetoplastid parasites (39–41). Growth conditions 1, 2, and 3 differed regarding the addition of dialyzed, regular, or no FBS, respectively. Condition 4 contained neither the supplements nor FBS. Condition 5 lacked additional hypoxanthine, 6-biopterin, and biotin as supplements but contained additional proline (Fig. S3a). Although parasites were viable under all growth conditions tested (Fig. S3b and c), serum-free conditions 3 and 4 resulted in atypical parasite morphologies that resembled stationary parasites. Growth was similar in dialyzed and regular FBS (Fig. S3c). We therefore chose growth condition 5 with dialyzed FBS to analyze the passage-dependent incorporation for heavy [^13^C]_6_-l-arginine and [^13^C]_6_-l-lysine. Mass spectrometry of whole-cell lysates revealed high incorporation efficiencies (with heavy-to-light ratios for all identified tryptic peptides of around 13) in the L. tarentolae proteome after two or three passages (Fig. S3d). We therefore opted for these conditions to determine the redox interactome of *Lt*Erv. In summary, based on a modified protocol for L. donovani (35), we identified a suitable medium and established a SILAC protocol for L. tarentolae.

### Mic20 and all but one typical twin Cx_3,9_C substrates are absent in the redox interactome of *Lt*Erv-His_8_.

**FIG 2:**
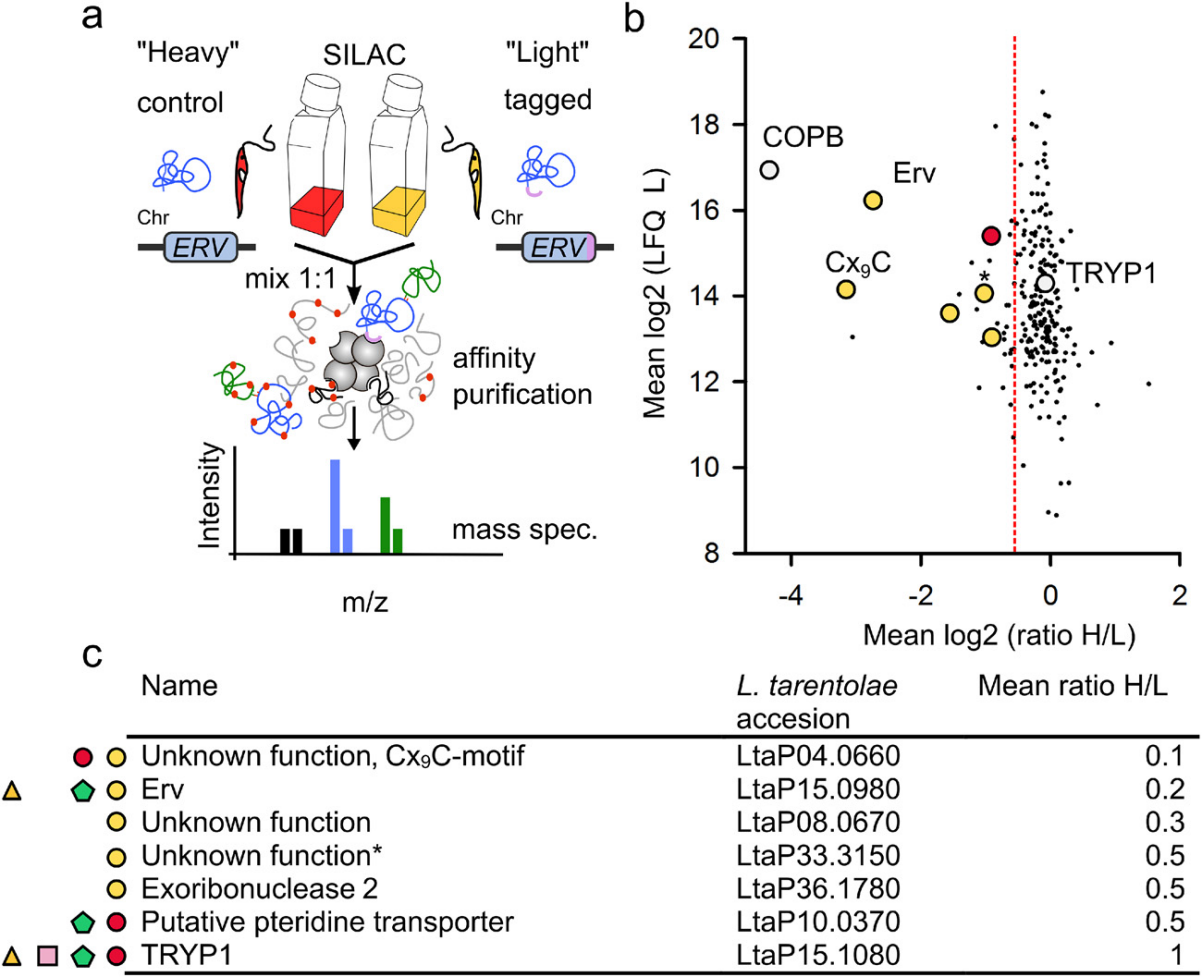
The redox interactome of *Lt*Erv-His_8_. (a) Design of the experiment, including SILAC, denaturing nonreducing affinity chromatography, and quantitative mass spectrometry. (b) Quantitative assessment of potential interaction partners of *Lt*Erv-His_8_. Proteins were classified as enriched if their 95% confidence interval exceeded Tukey’s outlier criterion, which was applied to the reference population (as indicated by the dotted red line). Significantly enriched candidates are highlighted in yellow. Homologues of a putative pteridine transporter and the 2-Cys peroxiredoxin TRYP1 were also detected in previous studies and are highlighted in red and gray, respectively. (c) Summary of the candidate proteins and their predicted function, accession number, and mean fold enrichment. Significantly enriched candidates are labeled with a yellow circle. Proteins with a red circle and a green pentagon were also found in the *Tb*Erv1 depletome and *Tb*ATOM40 importome, respectively (24). Proteins with a pink square and a yellow triangle were also detected in a previous pulldown with *Tb*Erv1 (29) and the redox interactome of *Lt*Erv^ΔKISS^-His_8_, respectively.

Depending on the presence or absence of a Mia40 replacement in kinetoplastid parasites, *Lt*Erv could transiently form a covalent disulfide bond either with the Mia40 replacement or with a variety of IMS substrates that contain a twin Cx_3,9_C motif. To identify the covalent interaction partner(s), we performed SILAC experiments in combination with denaturing Ni-nitrilotriacetic acid (NTA) affinity chromatography and mass spectrometry (Fig. 2a; Data set S1). Cells with *Lt*Erv-His_8_ were grown in light medium, and the parental line with wild-type *Lt*Erv was grown in heavy medium. Both cell lines were mixed 1:1 before cell lysis and subsequent analysis. *N*-ethylmaleimide (NEM) was added before cell lysis and during the purification process to block thiols and to prevent thiol-disulfide exchange reactions. More than 500 proteins were detected in the eluates from five biological replicates. Candidates were selected from peptides that were present in at least three of the five replicates. As expected for the rather harsh denaturing purification conditions, only a few proteins were enriched (Fig. 2b). The candidate Mic20 was absent in the eluates (Data set S1), although it was detected in total cell lysates from L. tarentolae. Furthermore, a previous *Tb*Erv1 pulldown experiment identified highly concentrated proteins, including a Prx1-type 2-Cys peroxiredoxin (TRYP1), as potential interaction partners (29). A homologue of this protein (LtaP15.1080) was found but was not enriched (Fig. 2b and c). Based on the statistical criteria outlined in Materials and Methods, only five of the identified proteins were classified as enriched—the bait *Lt*Erv-His_8_, three proteins with unknown function, and a putative exoribonuclease (Fig. 2c; Data set S1). The enrichment of the cysteine-rich putative beta prime COPB protein differed extremely between the experiments and was, therefore, not significant. Although twin Cx_3,9_C substrates were identified in total cell lysates, only one of the enriched candidates (LtaP04.0660, new GenBank number GET85674.1 [42]) has a typical twin Cx_3,9_C-motif. BLAST searches revealed that LtaP04.0660 and its twin Cx_9_C-motif are conserved among kinetoplastida. Homologues of LtaP04.0660 with a twin Cx_9_C-motif are also found in selected opisthokonts (excluding yeast) and very few amoebozoa but in no other major group of eukaryotes (Fig. S4a). MitoProII, CCTOP, and HHPRED (43) predicted a cleavable mitochondrial targeting sequence, a transmembrane segment at position 56 to 76, and a coiled-coil-helix-coiled-coil-helix domain (Fig. S4b). Thus, LtaP04.0660 shares many similarities with Mia40 from yeast, although it lacks the CPC active-site motif. Candidate LtaP08.0670 is probably a false-positive hit because it has no cysteine residue or mitochondrial targeting sequence. Candidate LtaP33.3150 has two predicted transmembrane segments and 13 cysteine residues, including an unusual twin Cx_9_Cx_3_C motif, but is not conserved among kinetoplastida. Hence, we excluded it as a candidate for a Mia40 replacement. In summary, Mic20 and all typical twin Cx_3,9_C substrates except for LtaP04.0660 were absent in the redox interactome of LtErv-His_8_.

### Redox treatments do not result in the accumulation of disulfide-bridged *Lt*Erv.

One plausible explanation for the small number of candidate proteins from the *Lt*Erv-His_8_ redox interactome could be short-lived mixed disulfide bond(s) between potential substrates or a Mia40 replacement and *Lt*Erv. We therefore tested different concentrations of NEM and *S*-methyl-methanethiosulfonate (MMTS) as alternative thiol-blocking agents and analyzed the formation of disulfide bonds between *Lt*Erv and other proteins by nonreducing SDS-PAGE and Western blot analysis (Fig. S5a and b). Furthermore, we treated wild-type cells for different periods with either the disulfide-inducing agent diamide (44) or DTT as a reducing agent before thiols were blocked with NEM (Fig. S5c and d). In another set of experiments, we added a cell lysis step with 5% trichloroacetic acid (TCA) on ice to quench thiolate groups and to prevent thiol-disulfide exchange reactions (Fig. S5e and f). However, none of the approaches with different thiol-blocking or redox agents as well as with or without TCA treatment led to an accumulation of mixed disulfides between *Lt*Erv and other proteins.

Next, we transfected wild-type L. tarentolae parasites with a set of plasmid pX-derived constructs that encode *Lt*Erv or *Lt*Erv^C63S^ with or without an N-terminal His_8_-tag for potential pulldown studies. Inactive His_8_-*Lt*Erv^63^ was selected as a putative trapping mutant because it cannot resolve disulfide bonds that are formed by the distal shuttle cysteine pair (28). However, the presence of large amounts of His_8_-*Lt*Erv or His_8_-*Lt*Erv^C63S^ did not result in the accumulation of mixed disulfides with other proteins (Fig. S6). In summary, neither redox treatments nor the presence of episomally encoded *Lt*Erv^C63S^ led to optimized trapping conditions for subsequent affinity chromatography.

### The shuttle cysteine motif of *Lt*Erv is crucial for parasite survival.

**FIG 3:**
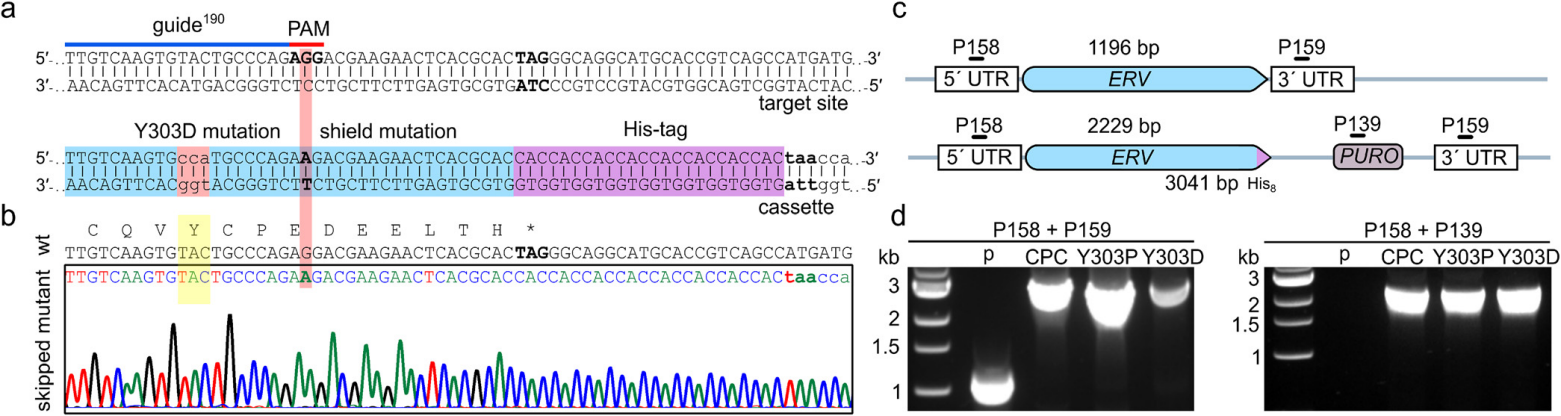
Mutations encoding altered shuttle cysteine motifs are skipped during homologous recombination. (a) Exemplary point mutation and tagging of *LTERV^Y303D^-His_8_*. A guide sequence was selected to introduce a double-strand break within the coding sequence for the CQVYC shuttle cysteine motif (top). The double-strand break was repaired by homologous recombination with a cotransfected donor DNA that encodes a mutation of the tyrosine codon in the 3′-homology region, a silent mutation of the PAM sequence (shield mutation), a His_8_-tag, and a puromycin resistance cassette followed by the 5′-homology region (bottom). (b) Section of a Sanger sequencing chromatogram of a representative PCR amplicon from homozygous puromycin-resistant parasites. The desired point mutations were skipped, in contrast to the successful shield mutation, tagging, and insertion of the puromycin resistance cassette. (c) Schematic representation of the wild-type and mutant *LTERV* loci on top and at the bottom, respectively. Only one locus is shown for diploid parasites. Primer names, binding sites, and expected product sizes from analytical PCRs are indicated. (d) Genotyping of the parental strain (p) and of representative clones that were obtained after selection with puromycin following transfection with donor DNA that encodes either *LTERV^CPC^-His_8_* (CPC), *LTERV^Y303P^-His_8_* (Y303P), or *LTERV^Y303D^-His_8_* (Y303D). Analytical PCRs with the indicated primers from panel c confirmed the homologous recombination and integration of the resistance cassette.

In order to perform *in vivo* structure-function analyses and to enrich potential disulfide-bonded substrates or a Mia40 replacement, we generated homozygous mutant cell lines for *LTERV*. First, we tried to modify the cysteine-intervening sequence within the CQVYC motif of the distal shuttle cysteine pair to slow down dithiol-disulfide exchange reactions. A similar kinetic trapping approach led to the identification of novel substrates of protein disulfide isomerase (PDI) (45). We therefore used the CRISPR-Cas9 system to introduce a chromosomal double-strand break near the codon for the intervening residue Tyr^303^ in both *LTERV* gene copies and provided a DNA repair fragment with homology regions that flank the tagged mutated 3′ end of *LTERV* and an antibiotic resistance cassette (Fig. 3a). Using this approach, we attempted to replace Tyr^303^ with either proline or aspartate to perturb the conformation or charge of the intervening sequence. Furthermore, we tried to replace the CQVYC motif with a constraint CPC motif (the active site motif of Mia40, which is a rather slow redox enzyme [18, 46–48]). Although cell lines were obtained for each mutant after clonal selection on agar plates, sequence analysis revealed that the tag but not the desired point mutation was introduced (Fig. 3b). PCR analyses confirmed the expected integration of the antibiotic resistance cassette (Fig. 3c and d). Thus, only parasites that skipped the desired mutation but included the protospacer-adjacent motif (PAM) mutation, tag, and antibiotic resistance cassette during homologous recombination were selected. To prevent such recombination events, we generated two template plasmids with recodonized *LTERV* (pPLOT-*His_8_*-*ERV_recodon_*-*His_8_-PURO* and pPLOT-*His_8_*-*ERV_recodon_*-*His_8_-BLAST*) to amplify the DNA repair fragments. No colonies were obtained for parasites with *Lt*Erv^Y303P^-His_8_, *Lt*Erv^Y303D^-His_8_, or *Lt*Erv^CPC^-His_8_ after simultaneous selection with puromycin and blasticidin in two independent biological replicates. In summary, the skipped mutations and the lack of viable parasites using mutated recodonized *LTERV* for DNA repair altogether indicate that Tyr^303^ and the shuttle cysteine pair of *Lt*Erv exert essential functions in L. tarentolae.

### Cys^17^ of *Lt*Erv is dispensable for parasite survival.

**FIG 4:**
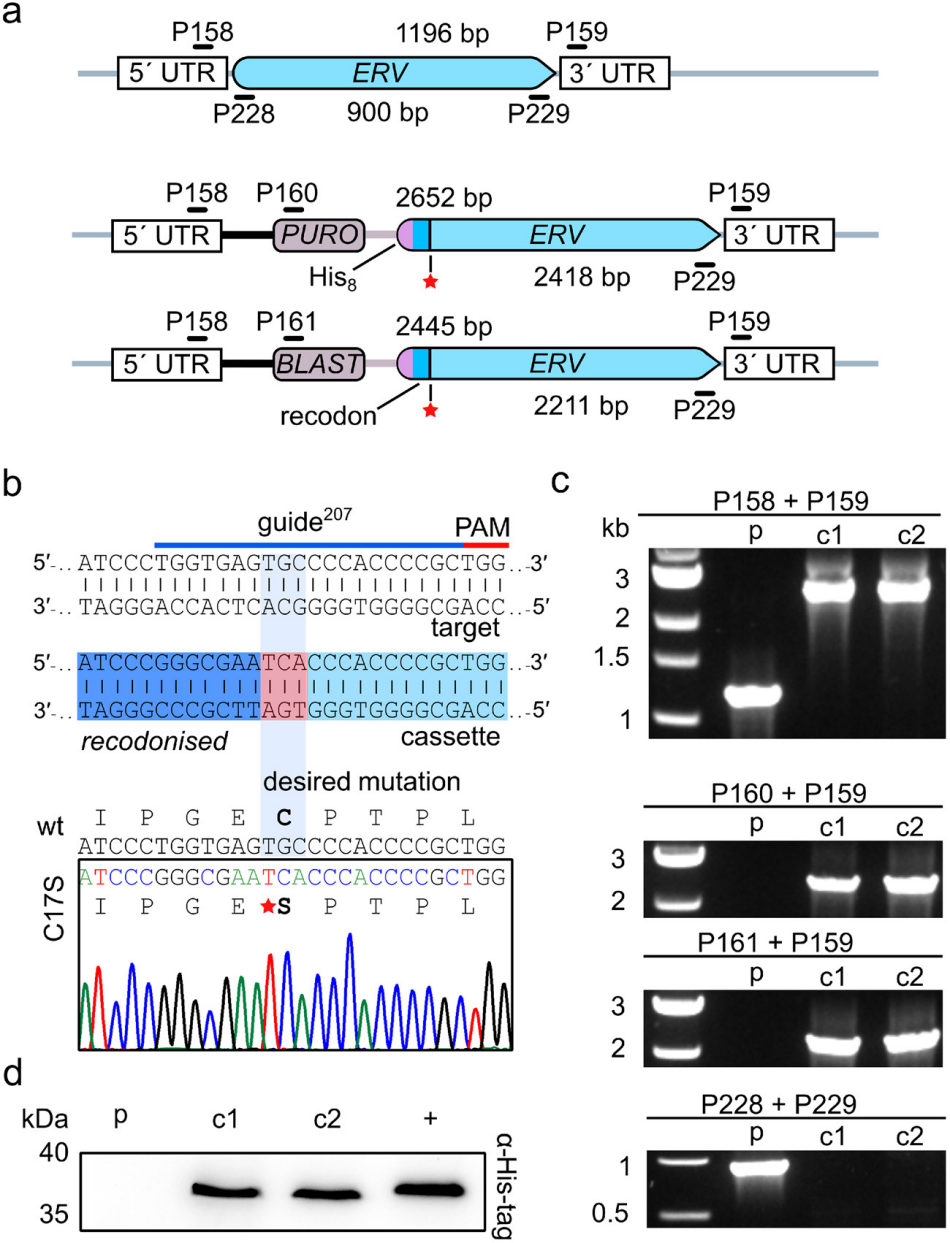
Generation and validation of L. tarentolae strains with chromosomally encoded His_8_-*Lt*Erv^C17S^. (a) Schematic representation of the loci for wild-type *LTERV* (top) and mutant *HIS_8_-LTERV^C17S^* with antibiotic resistance cassettes against puromycin or blasticidin (bottom). Primer binding sites and expected product sizes from analytical PCRs are indicated. (b) Guide sequence and sections of the donor DNA sequence and a Sanger sequencing chromatogram from a representative PCR amplicon of a homozygous clone encoding His_8_-*Lt*Erv^C17S^. The point mutation was introduced between the recodonized 5′ fragment of *LTERV* (dark blue) and the 5′-homology region (light blue). (c) Genotyping of the parental strain (p) and of two clonal strains (c1 and c2) that were obtained after selection with puromycin and blasticidin. Analytical PCRs with the indicated primers from panel a confirmed the homologous recombination, the integration of the resistance cassettes, and the loss of wild-type *LTERV*. (d) Western blot analysis of clones c1 and c2 with His_8_-*Lt*Erv^C17S^ with a calculated molecular mass of 35.7 kDa. The parental strain (p) and a strain with *Lt*Erv-His_8_ (+) served as negative and positive controls, respectively.

Second, we addressed a potential physiological function of clamp residue Cys^17^, which was previously shown to render *Lt*Erv incompatible with the oxidative protein folding machinery in yeast (32). We therefore introduced a point mutation in *LTERV* using the CRISPR-Cas9 system and DNA repair fragments with an antibiotic resistance cassette and a tagged recodonized 5′ fragment of *LTERV* before the mutated 3′-homology region (Fig. 4a). Two clonal cell lines were further characterized after selection on agar plates with puromycin and blasticidin. Sequence analysis revealed that the desired point mutation was introduced right after the recodonized 5′ fragment of *LTERV* (Fig. 4b). PCR analyses excluded ectopic recombination events and confirmed the correct integration of the antibiotic resistance cassettes and the loss of wild-type *LTERV* (Fig. 4c). Successful N-terminal tagging of His_8_-*Lt*Erv^C17S^ was revealed by Western blot analysis (Fig. 4d). The N-terminal His_8_-tag and the replacement of Cys^17^ did not cause a growth defect in brain heart infusion (BHI) liquid medium compared to the parental strain (Fig. S7). In summary, clamp residue Cys^17^ is dispensable for parasite survival, and its replacement in combination with N-terminal His_8_-tagging of *Lt*Erv has no effect on parasite growth.

### The KISS domain influences the redox state of *Lt*Erv but is dispensable for parasite survival.

**FIG 5:**
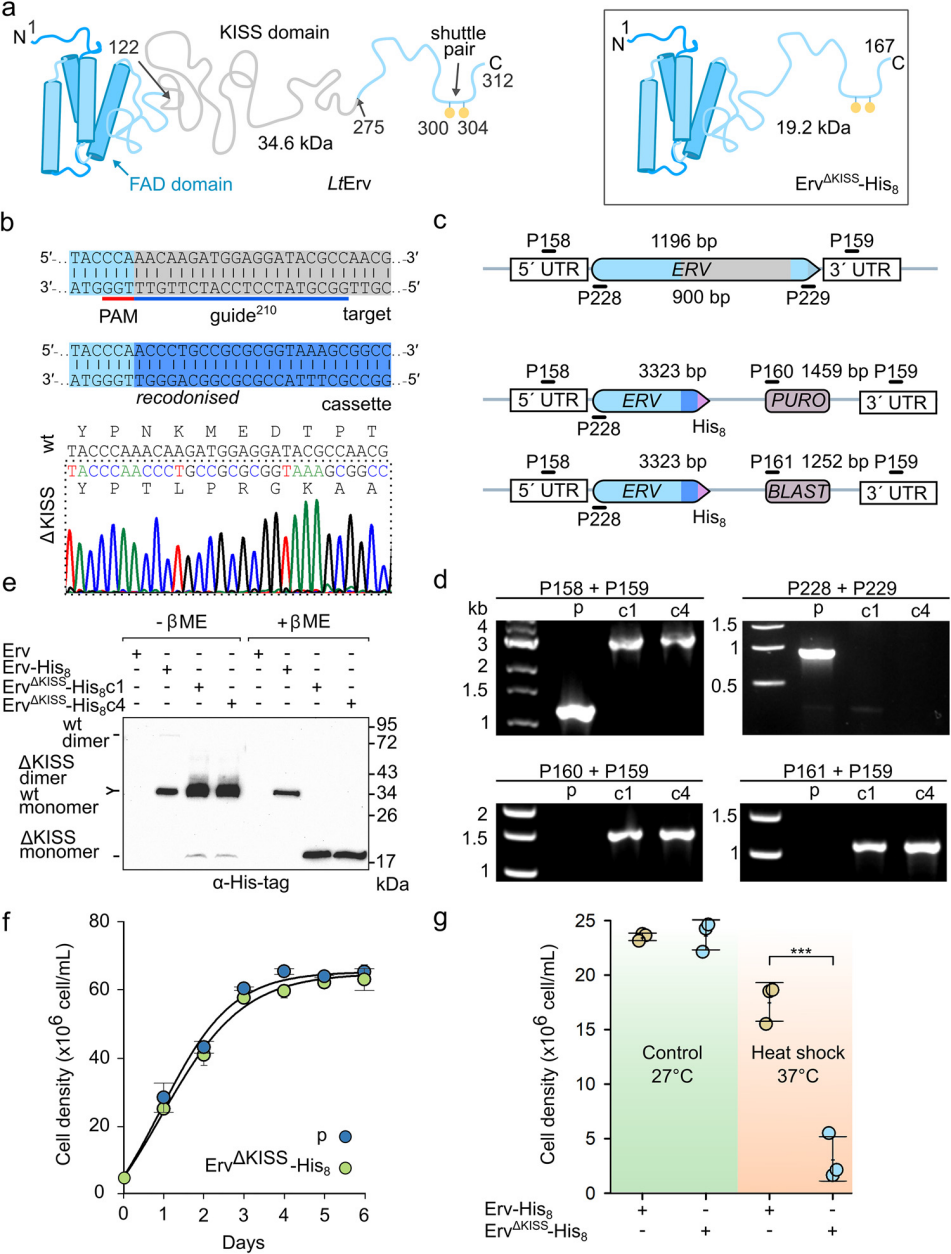
Generation and validation of L. tarentolae strains with chromosomally encoded *Lt*Erv^ΔKISS^-His_8_. (a) Schematic representation of wild-type *Lt*Erv with its N-terminal flavodomain, the KISS domain, and the C-terminal shuttle arm (left side) and *Lt*Erv^ΔKISS^-His_8_ with its fused N-terminal flavodomain and tagged C-terminal shuttle arm (right side). Amino acid positions and the calculated molecular masses are indicated. (b) Guide sequence and sections of the donor DNA sequence and a Sanger sequencing chromatogram from a representative PCR amplicon of a homozygous clone encoding *Lt*Erv^ΔKISS^-His_8_. A double-strand break was introduced at the 3′ end of the sequence that encodes the flavodomain (light blue) before the KISS domain (gray). Recodonized tagged *LTERV* (dark blue) was used as a donor DNA. (c) Schematic representation of the loci for wild-type *LTERV* (top) and mutant *LTERV^ΔKISS^-HIS_8_* with antibiotic resistance cassettes against puromycin or blasticidin (bottom). Primer binding sites and expected product sizes from analytical PCRs are indicated. (d) Genotyping of the parental strain and of two clonal strains (c1 and c4) that were obtained after selection with puromycin and blasticidin. Analytical PCRs with the indicated primers from panel d confirmed the homologous recombination, the integration of the resistance cassettes, and the loss of wild-type *LTERV*. (e) Western blot analysis of clones c1 and c4. Nonreducing and reducing SDS-PAGE ± β-mercaptoethanol (β-ME) revealed the conversion of most likely dimeric into monomeric *Lt*Erv^ΔKISS^-His_8_ with calculated molecular masses of 38.5 kDa and 19.2 kDa. The parental strain with wild-type *Lt*Erv and a strain with *Lt*Erv-His_8_ (with a calculated molecular mass of 35.7 kDa) served as negative and positive controls, respectively. (f) Growth curve analysis in BHI liquid medium of the parental strain with wild-type *Lt*Erv (p) and a strain with *Lt*Erv^ΔKISS^-His_8_. Data points represent the mean ± standard deviation from two independent biological replicates. (g) Heat shock of L. tarentolae strains with *Lt*Erv-His_8_ or *Lt*Erv^ΔKISS^-His_8_. Standard promastigote liquid cultures were split to an initial cell density of 5 × 10^6^ cells/ml and either incubated at 27°C or shifted overnight to 37°C without shaking. All cultures were subsequently allowed to recover at 27°C for 24 h before the cell densities were determined. Data points represent the mean ± standard deviation from three independent biological replicates. A *P* value of ≤ 0.001 (***) for heat-shocked parasites was calculated in SigmaPlot 13 using a one way analysis of variance (ANOVA).

Third, we addressed the physiological relevance of the KISS domain, which has no cysteine residue and makes up almost half of the total protein sequence of *Lt*Erv (Fig. 5a) (28). We therefore generated L. tarentolae cell lines that chromosomally encode C-terminally His_8_-tagged *Lt*Erv without residues 123 to 275 (*Lt*Erv^ΔKISS^-His_8_). In this protein, the FAD-binding domain is directly fused to the C-terminal arm with the distal shuttle cysteine pair (Fig. 5a). Using the CRISPR-Cas9 system, we introduced a chromosomal double-strand break near the codon for Tyr^122^ in both *LTERV* gene copies (Fig. 5b) and provided DNA repair fragments with homology regions that flank a tagged recodonized 3′ fragment of *LTERV* and an antibiotic resistance cassette (Fig. 5c). Two clonal cell lines were further characterized after selection with puromycin and blasticidin. PCR analyses of these clones confirmed the replacement of wild-type *LTERV* and the correct insertion of both antibiotic resistance cassettes. Furthermore, PCR analyses excluded ectopic recombination events and showed the complete loss of wild-type *LTERV* for both cell lines (Fig. 5d). Sequencing of the PCR amplicon for *LTERV^ΔKISS^-HIS_8_* confirmed the homozygous in-frame recombination event (Fig. 5b), and Western blot analysis showed the presence of the His-tagged protein in whole-cell lysates (Fig. 5e). However, in contrast to monomeric *Lt*Erv-His_8_, removal of the KISS-domain shifted the equilibrium in whole-cell lysates toward disulfide-bridged dimeric *Lt*Erv^ΔKISS^-His_8_ (or toward a heterodimer with the same mass). Although cell lines with *Lt*Erv^ΔKISS^-His_8_ did not display a growth defect in BHI liquid medium under standard growth conditions (Fig. 5f), removal of the KISS domain rendered parasites more sensitive to heat stress (Fig. 5g).

**FIG 6:**
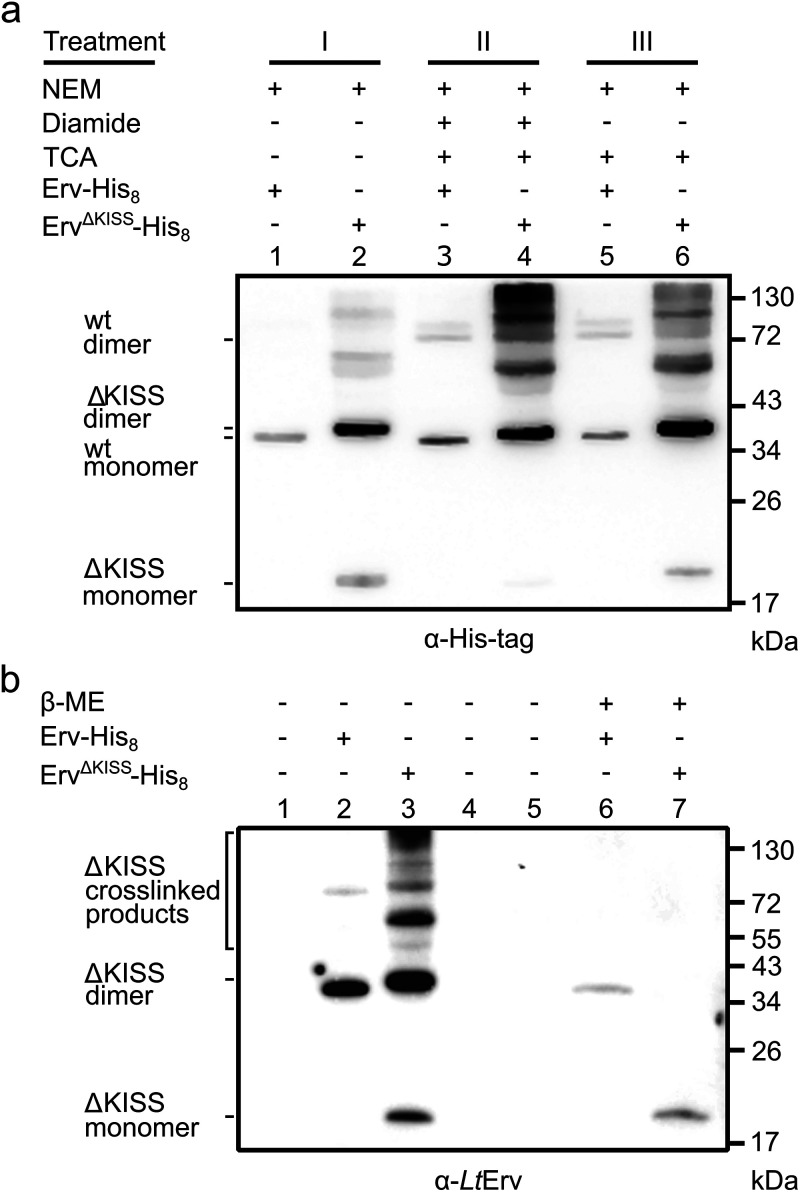
Chemical trapping of mixed *Lt*Erv^ΔKISS^-His_8_ disulfides. Western blot analysis of whole-cell lysates from L. tarentolae strains with *Lt*Erv^ΔKISS^-His_8_ or *Lt*Erv-His_8_. (a) Cells and lysates were treated as follows before protein separation by nonreducing SDS-PAGE and Western blot analysis against the His tag. In treatment I, NEM was added to intact cells and whole-cell lysates. In treatments II and III, intact cells were treated with or without diamide as an oxidant. TCA was added to lyse the cells and to quench reactive thiolates. NEM was added to the TCA pellet. Samples with *Lt*Erv-His_8_ with a calculated molecular mass of 35.7 kDa served as controls and revealed predominantly monomeric protein (wild-type [wt] monomer). Samples with *Lt*Erv^ΔKISS^-His_8_ revealed predominantly disulfide-bridged homodimeric protein (ΔKISS dimer) and mixed disulfide-bridged protein species with higher molecular masses. (b) Western blot analysis against *Lt*Erv following treatment II confirmed the conversion of most likely homodimeric *Lt*Erv^ΔKISS^-His_8_ with a calculated molecular mass of 38.5 kDa and mixed *Lt*Erv^ΔKISS^-His_8_ disulfides to reduced monomeric *Lt*Erv^ΔKISS^-His_8_ with a calculated molecular mass of 19.2 kDa.

To further enrich or stabilize disulfide-bonded proteins, we compared three different chemical trapping protocols for *Lt*Erv^ΔKISS^-His_8_ and *Lt*Erv-His_8_ (Fig. 6). Nonreducing SDS-PAGE and Western blot analysis confirmed the shift in redox equilibrium toward disulfide-bridged dimeric *Lt*Erv^ΔKISS^-His_8_ at about 38 kDa, in contrast to monomeric *Lt*Erv-His_8_ at approximately 36 kDa (Fig. 6a). More importantly, all three protocols and, in particular, treatment with TCA and/or diamide resulted in the accumulation of additional protein-protein disulfides for *Lt*Erv^ΔKISS^-His_8_, whereas *Lt*Erv-His_8_ remained unmodified except for a small fraction that formed homodimers. Western blot analysis following reducing SDS-PAGE resulted in the conversion of the protein disulfide patterns to a single band for monomeric *Lt*Erv^ΔKISS^-His_8_ around 19 kDa as expected (Fig. 6b). In summary, deletion the KISS domain (i) does not affect the viability of L. tarentolae under standard growth conditions, (ii) renders parasites more sensitive to heat stress, and (iii) stabilizes disulfide-bonded dimeric *Lt*Erv^ΔKISS^-His_8_ as well as additional mixed disulfides with other proteins.

### Mic20 and typical twin Cx_3,9_C substrates are also absent in the redox interactome of *Lt*Erv^ΔKISS^-His_8_.

**FIG 7:**
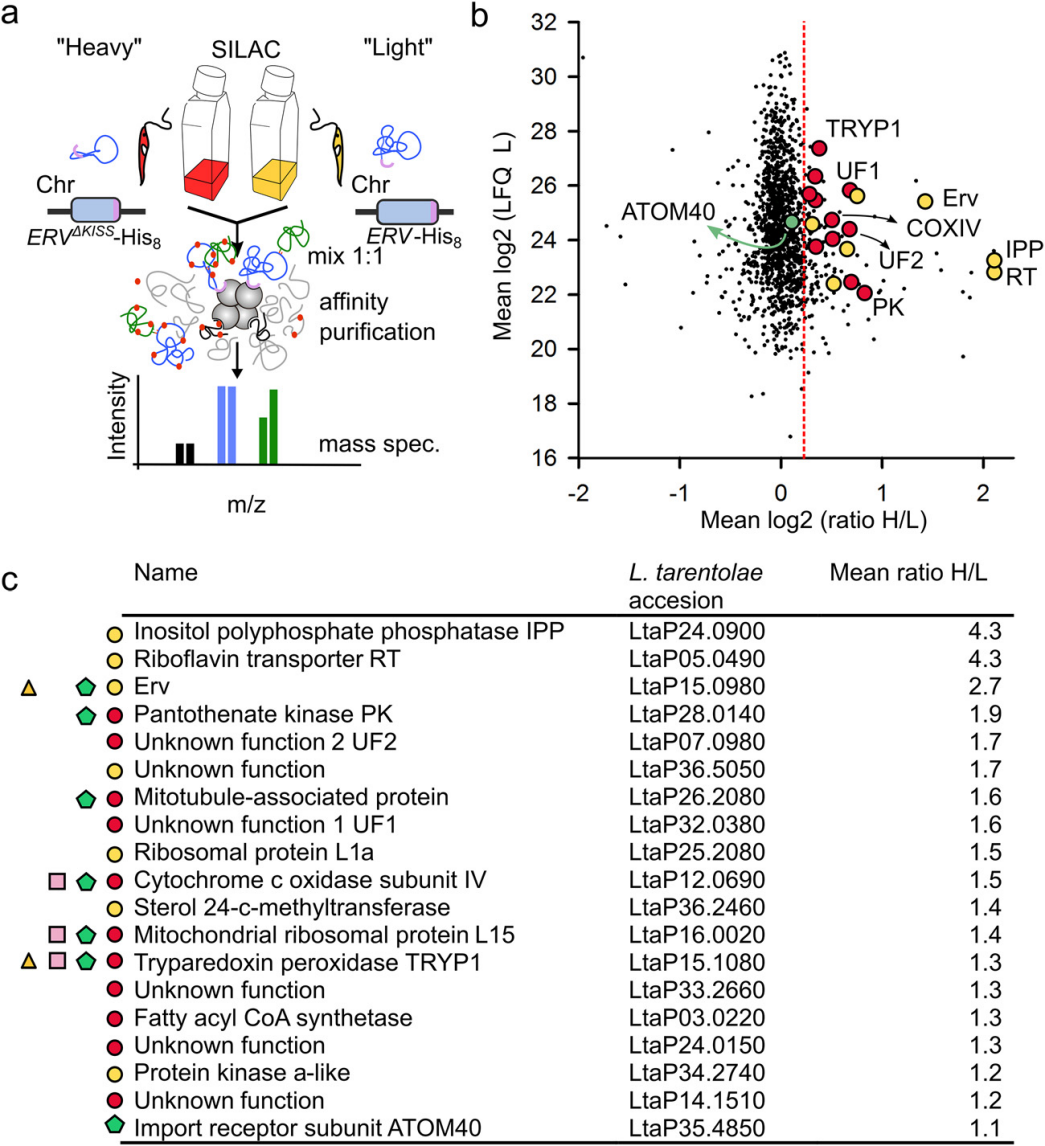
The redox interactome of *Lt*Erv^ΔKISS^-His_8_. (a) Design of the experiment, including SILAC, denaturing nonreducing affinity chromatography, and quantitative mass spectrometry. (b) Quantitative assessment of potential interaction partners that were enriched for *Lt*Erv^ΔKISS^-His_8_ compared to *Lt*Erv-His_8_. Proteins were classified as enriched if their 95% confidence interval exceeded Tukey’s outlier criterion, which was applied to the reference population (as indicated by the dotted red line). Significantly enriched candidates and homologues that were also detected in the *Tb*Erv1 depletome are highlighted in yellow and red, respectively. (c) Summary of the candidate proteins and their predicted function, accession number, and mean fold enrichment. True candidates are labeled with a yellow circle. Proteins with a red circle and a green pentagon were also found in the *Tb*Erv1 depletome and *Tb*ATOM40 importome, respectively (24). Proteins with a pink square and a yellow triangle were also detected in a previous pulldown with *Tb*Erv1 (29) and the redox interactome of *Lt*Erv-His_8_, respectively.

The accumulation of mixed disulfides for *Lt*Erv^ΔKISS^-His_8_ allowed us to perform another SILAC experiment to analyze the identity of the interaction partners and a potential enrichment of substrates or a Mia40 replacement. Cells with *Lt*Erv^ΔKISS^-His_8_ were grown in heavy medium, and cells with *Lt*Erv-His_8_ were grown in light medium as a control (Fig. 7a). Both cell lines were processed as described above. Eluates from affinity chromatography were concentrated, reduced, alkylated, and digested with trypsin. Mass spectrometry from biological duplicate experiments led to the identification of 880 proteins. Again, a homologue of *Tb*Mic20 was not identified, and there was also no classical substrate with a twin Cx_3,9_C motif among the significantly enriched candidates (Fig. 7b and c; Data set S2). A putative monocarboxylate or riboflavin transporter (RT) and a putative inositol polyphosphate phosphatase (IPP) were the most enriched proteins. *Lt*Erv^ΔKISS^-His_8_ itself was also enriched compared to *Lt*Erv-His_8_. This might point to an upregulation as a compensatory mechanism for the altered redox state and is consistent with the Western blot data in Fig. 6. A moderately enriched candidate protein with unknown function 1 (UF1, LtaP32.0380) is conserved in kinetoplastid parasites and has a *C*HAY*C*R*C*SY*C*-motif close to the N terminus and an internal PDI-type *C*GH*C*-motif, suggesting a dithiol:disulfide oxidoreductase activity (Fig. S8a). Furthermore, the moderately enriched homologue of the identified 270-kDa protein with unknown function 2 (UF2, LtaP07.0980) (Fig. 7b and c) was the most depleted protein in *Tb*Erv1 knockdown parasites (24), is conserved in kinetoplastid parasites, and has a CxC motif similar to that of Mia40 (Fig. S8b) in combination with an unusual CCx_8_C+Cx_8_CC motif. The Prx1-type 2-Cys peroxiredoxin LtaP15.1080/LtaP15.1060 (GenBank accession number GET87294.1), which was also identified in the other pulldown experiments with *Lt*Erv-His_8_, was highly abundant but only slightly enriched. Its T. brucei homologue TRYP1 (encoded by the duplicated genes Tb927.9.5770 and Tb927.9.5860) was also detected by mass spectrometry in *Tb*Erv1 pulldown studies (29) and was 1.34-fold enriched (not depleted) in mitochondrial fractions from *Tb*Erv1 knockdown parasites (24). The identified homologue of the cytochrome *c* oxidase subunit IV LtaP12.0690 was also detected in the *Tb*Erv1 pulldown studies and was 0.79-fold depleted in *Tb*Erv1 knockdown parasites (24, 29). In summary, our redox interactome of *Lt*Erv^ΔKISS^-His_8_ led to the identification of neither a Mic20 homologue nor typical substrates with twin Cx_3,9_C-motifs but points toward alternative interaction candidates such as LtaP32.0380, LtaP07.0980, or a 2-Cys peroxiredoxin. These candidates could replace Mia40 in kinetoplastid parasites and/or exert alternative functions that are linked to *Lt*Erv.

## DISCUSSION

**FIG 8:**
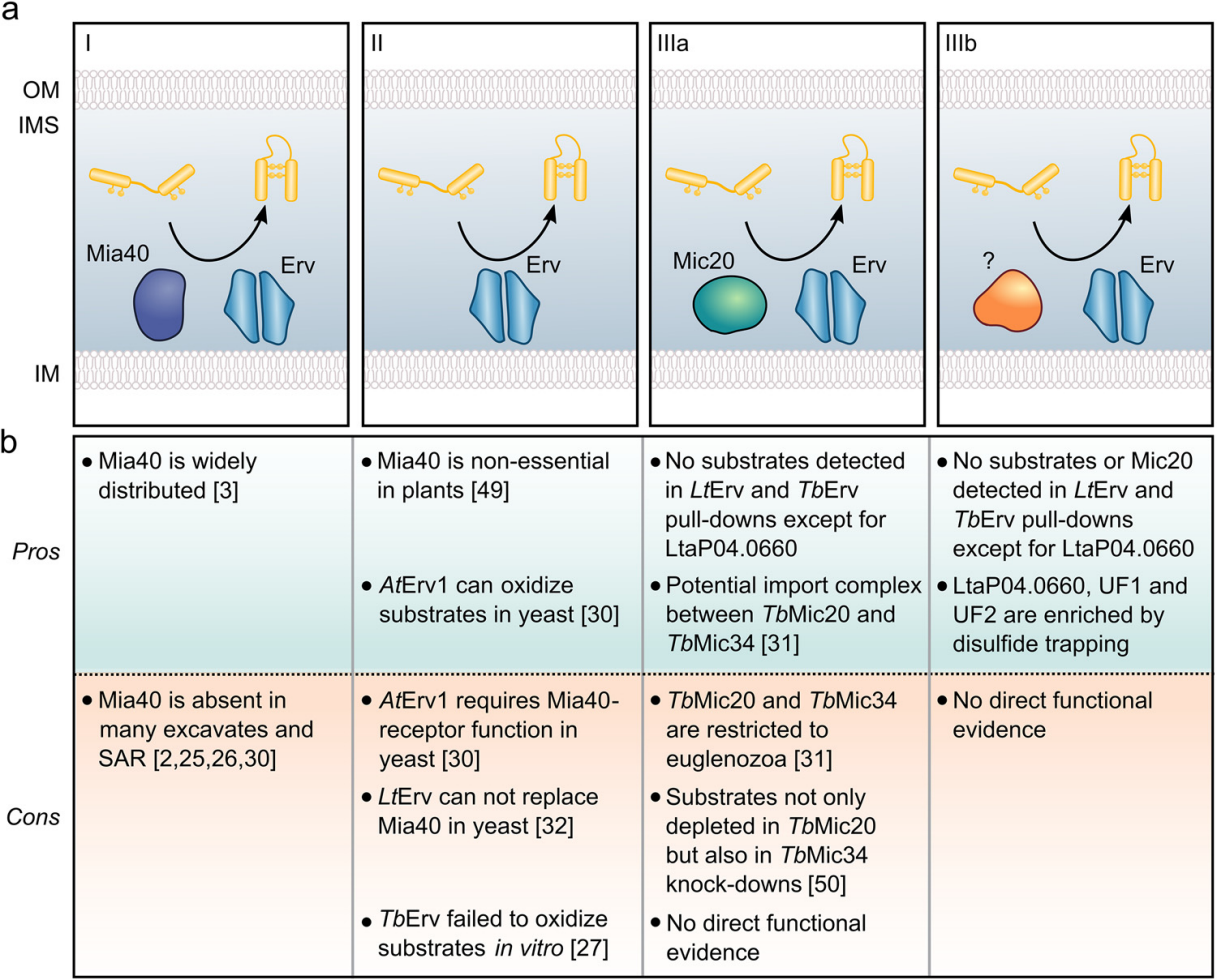
Overview of current models of oxidative protein folding in the mitochondrial intermembrane space. (a) Three conceptually different models can be discriminated. (I) The classical two-component system consisting of Mia40 and Erv1 is found in yeast and other opisthokonts, as well as plants. (II) A simplified single-component system has been suggested, but its physiological functionality remains to be demonstrated. (III) The physiological functionality of alternative two-component systems with a Mia40 receptor and/or oxidoreductase replacement also remains to be demonstrated. (b) Pros and cons of the different models in panel (a).

We previously suggested three different scenarios for the evolution of the Mia40/Erv system and the oxidative protein-folding pathway in the IMS: primitive eukaryotes contained the ancestors of either (i) Erv and Mia40, (ii) Erv alone, or (iii) Erv and an unknown alternative protein that functionally replaced Mia40 (2). The likelihood of each scenario is usually discussed based on hypotheses for current model systems (Fig. 8). Scenario i seems unlikely because of basic and advanced *in silico* analyses that did not reveal a Mia40 homologue in several eukaryotic lineages (2, 25, 26). A pulldown experiment in T. brucei using a *Tb*Erv1 antibody also did not reveal a Mia40 replacement, and this absence of evidence was interpreted in favor of scenario ii (29). Furthermore, experiments in Arabidopsis thaliana suggested that Mia40 could be dispensable for mitochondrial protein import in plants (49). These results can be interpreted either as a representative case for scenario ii, i.e., *At*Erv1 can exert its function without *At*Mia40, or as a combination of scenarios i and iii in accordance with two redundant protein import systems in plants. In order to address these possibilities, Peleh et al. conducted a plasmid shuffling experiment in yeast and showed that *At*Erv1 can complement the loss of *Sc*Erv1 in the presence of redox-inactive *Sc*Mia40^SPS^ (30). The authors interpreted the results as a proof for stepwise evolution based on the single-component scenario ii. The key experiment, however, required the presence of *Sc*Mia40^SPS^, which still served as an essential receptor for the incoming cysteine-containing substrates. Hence, the result from the plasmid-shuffling experiment should, in our opinion, be interpreted as a special case of the two-component scenario iii with Erv and an essential (in this case redox-inactive) receptor protein. (Of note, our denaturing purification protocols were designed to catch disulfide-bridged interaction partners and would therefore miss a Mia40^SPS^ replacement that functions as a redox-inactive receptor). If scenario ii was correct, one would also expect that canonical IMS substrates with twin Cx_3,9_C motifs are identified as direct interaction partners of Erv homologues by nonreducing SDS-PAGE, Western blot analysis, or mass spectrometry. However, neither previous studies on the T. brucei homologue *Tb*Erv1 (29) nor our studies on *Lt*Erv revealed such interactions except for LtaP04.0660 (the unknown protein with a twin Cx_9_C motif) in the *Lt*Erv-His_8_ redox interactome and LtaP07.0980 (UF2 with an unusual CCx_8_C+Cx_8_CC motif) in the *Lt*Erv^ΔKISS^-His_8_ redox interactome. Furthermore, recombinant *Tb*Erv1 was unable to directly oxidize a small Tim *in vitro* (27), and *Lt*Erv could also not complement the loss of *Sc*Mia40 in yeast (32). Scenario iii is also in accordance with a recent study that identified the thioredoxin-like MICOS component Mic20 as a Mia40-replacement candidate in kinetoplastid parasites based on similar RNAi knockdown phenotypes for *Tb*Mic20 and *Tb*Erv1 (24, 31). However, *Tb*Mic20 was absent in *Tb*Erv1 coimmunoprecipitation experiments (29), and proteins with twin Cx_3,9_C-motifs were also depleted by the knockdown of the MICOS component *Tb*Mic34 (50). Although the latter depletion could be an indirect/secondary effect due to the depletion of *Tb*Mic20 in the *Tb*Mic34 RNAi experiment, there is currently no direct evidence for the role of *Tb*Mic20 and/or *Tb*Mic34 as a Mia40-like protein thiol:disulfide oxidoreductase or protein receptor. Whether *Tb*Mic20 and/or *Tb*Mic34 directly interact with *Tb*Erv1 and altogether catalyze disulfide-bond formation remains to be shown. Another interesting aspect is that Mic20 homologues are absent in nonrelated protist lineages that lack Mia40 so that we would still not understand how Erv homologues exert a function in oxidative protein folding in these organisms. As a relative of T. brucei, L. tarentolae has apparently two Mic20 homologues (LtaP26.1460 and LtaP33.1480; however, LtaP33.1480 might be an incorrect redundant annotation for the homologue deposited under GenBank accession number GET91873.1 [42]). Although LtaP26.1460 was detected by mass spectrometry in lysate controls, both proteins were absent in the *Lt*Erv-His_8_ and *Lt*Erv^ΔKISS^-His_8_ interactomes. Whether the candidates from our redox interactomes are essential and play a role in oxidative protein folding or exert another Erv-dependent function remains to be analyzed. According to high-throughput RNAi studies in T. brucei (51) that are listed in TriTrypDB (52), knockdown of Tb927.10.14490, which is the homologue of LtaP32.0380 from Fig. 6, causes an extreme loss of fitness, whereas knockdowns of the T. brucei homologues of LtaP04.0660 and LtaP07.0980 (Tb927.9.7980 and Tb927.8.830, respectively) can result in either a gain or loss of fitness depending on the investigated developmental stage. In summary, our data support neither a direct substrate oxidation by *Lt*Erv in accordance with scenario ii nor a role of Mic20 in oxidative protein folding in accordance with scenario iii but rather point toward alternative Mia40-replacement candidates or interaction partners, including the uncharacterized proteins LtaP04.0660, LtaP32.0380, and LtaP07.0980.

A possible bottleneck of our study could be a rapid substrate turnover. While mixed disulfides between *Sc*Mia40 and *Sc*Erv1 or incoming substrates are long-lived and detectable by Western blot analysis (18, 48, 53), *Lt*Erv seems to form short-lived mixed disulfide bonds that are difficult to detect. The addition of small peptide tags to the N or C terminus of *Lt*Erv did not impair parasite growth, in contrast to previous attempts to tag *Lt*Erv with bulky mCherry (33). Parasites with an altered shuttle pair CQVYC-motif to slow down dithiol-disulfide exchange reactions were not viable. We therefore used *Lt*Erv^ΔKISS^-His_8_ to enrich candidate interaction partners, some of which are conserved in kinetoplastid parasites and have cysteine motifs that point toward a redox activity. This includes LtaP32.0380 with a PDI-like CGHC-motif and LtaP07.0980 with a Mia40-like CxC-motif. Another interesting protein is the Prx1-type 2-Cys peroxiredoxin LtaP15.1080. Its homologue TRYP1 has been identified in previous *Tb*Erv1 pulldown experiments (29). Prx1-type 2-Cys peroxiredoxins are ubiquitous, highly abundant enzymes that exert functions as hydroperoxidases, redox sensors, and chaperones (54–56). In addition, mammalian PrxIV was shown to assist oxidative protein folding in the endoplasmic reticulum by introducing protein disulfide bonds in PDI (57, 58). Some dually localized opisthokont peroxiredoxin homologues, including mammalian PrxIII and PrxIV (59) as well as yeast Prx1 (60), were also suggested to be present in the IMS. Furthermore, alternative translation initiation was shown to target the yeast peroxidase *Sc*GPx3 to the IMS, where it interacts with *Sc*Mia40 (61). Our quantitative interactomes with *Lt*Erv-His_8_ and *Lt*Erv^ΔKISS^-His_8_ did not reveal a significant enrichment of LtaP15.1080. Whether peroxidases exert a conserved role in oxidative protein folding in mitochondria or are just false-positive hits because of their high reactivity and concentration remains to be analyzed in more detail.

It is tempting to hypothesize that the KISS domain and/or clamp residue Cys^17^ plays a role in mitochondrial protein import (28, 31, 32). For example, the KISS domain might interact with substrate proteins or Mic20 or might be relevant for the *Sc*Mia40-independent import of *Lt*Erv in yeast and *Leishmania* mitochondria. However, neither Cys^17^ nor the KISS domain are essential, and replacement of Cys^17^ or removal of the KISS domain had no effect on the growth of L. tarentolae promastigotes in BHI medium. One detectable effect for *Lt*Erv^ΔKISS^-His_8_ was the stabilization of most likely dimeric Erv-SS-Erv as well as mixed disulfides with other proteins. Hence, the KISS domain supports the efficient reduction of *Lt*Erv homodimers and *Lt*Erv substrates. It remains to be analyzed whether the accumulation of protein disulfides was caused by an altered redox potential of the shuttle cysteine residues or by altered *Lt*Erv conformations that slow down the Cys^66^-dependent reduction of the disulfide bond between residues Cys^304^′ and Cys^63^ or the Cys^304^-dependent reduction of mixed disulfides (28). The decreased tolerance of promastigotes with *Lt*Erv^ΔKISS^-His_8_ toward heat stress might indeed point to alternative, destabilized protein conformations. An alternative interpretation is that the KISS domain might have a chaperone function that becomes relevant at elevated temperatures. Furthermore, it is interesting to note that L. tarentolae was tolerant regarding the indirect manipulation of the redox properties of *Lt*Erv by removing the KISS domain, whereas subtle direct modifications of the CQVYC motif were lethal. Yeast, for example, was shown to be much more robust and to tolerate modifications of the N-terminal shuttle-cysteine motif of *Sc*Erv1 (32).

In conclusion, our *in vivo* structure-function analyses revealed that, in contrast to the shuttle cysteine motif, neither the KISS domain nor residue Cys^17^ of *Lt*Erv are essential for L. tarentolae viability. Removal of the KISS domain renders parasites sensitive toward heat stress. Furthermore, we identified two alternative sets of candidate interaction partners of *Lt*Erv, including the uncharacterized proteins LtaP04.0660, LtaP32.0380, and LtaP07.0980, whereas the homologue of *Tb*Mic20 could not be enriched. Although our data rather contradict a direct substrate oxidation, it remains to be shown whether a Mia40 replacement is or was a prerequisite for oxidative protein folding in the IMS of kinetoplastid parasites or primitive eukaryotes, respectively.

## MATERIALS AND METHODS

### Cell culture.

L. tarentolae Parrot TarII/UC strain promastigotes were cultured at 27°C in ventilated tissue culture (TC) flasks in an upright position on a Heidolph Rotamax 120 shaker at 50 rpm in 10 ml brain heart infusion (BHI) medium that was supplemented with 10 μg/ml hemin. Cultures were diluted 1:10 to 1:20 with fresh medium to maintain growth in the mid-log phase at ≤5 × 10^7^ cells/ml. Cell densities were determined in a hemocytometer following the immobilization of parasites by mixing an aliquot of the culture with one to three volumes of fixation solution containing 10% (vol/wt) paraformaldehyde, 0.15 M NaCl, and 15 mM trisodium citrate, pH 7.4. For SILAC experiments, L. tarentolae promastigotes were cultured based on a modified protocol for L. donovani (35). Parasite growth in different media was analyzed for medium 199 (M199) with 10% heat-inactivated FBS (Gibco), 40 mM HEPES, pH 7.4, and 10 μg/ml hemin. This medium was further supplemented with or without 2 mM l-glutamine, 5.2 mM l-proline, 0.1 mM adenosine, 0.1 mM hypoxanathine, 10 μM folic acid, 40 μM biotin, and/or 10 μM 6-biopterin as indicated. Subsequent SILAC experiments were performed with parasite cultures that were transferred from BHI medium to custom-made M199 without l-arginine and l-lysine (Caisson Laboratories, UT, USA; no. MDP02) containing 10% dialyzed heat-inactivated FBS, 40 mM HEPES, pH 7.4, 10 μg/ml hemin, 2 mM l-glutamine, 5.2 mM l-proline, 0.1 mM adenosine, and 10 μM folic acid. For light medium, 42 mg/liter l-arginine and 73 mg/liter l-lysine were added to supplemented M199. For heavy medium, 43.3 mg/liter [^13^C]_6_-l-arginine (Sigma-Aldrich; no. 643440) and 75.4 mg/liter [^13^C]_6_-l-lysine (Sigma-Aldrich; no. 643459) were added to supplemented M199. To label the proteome, parasites were first adapted in control light medium for 3 days. Promastigotes were subsequently passaged 1:20 in light or heavy medium every third day. Parasites were harvested after passages 2 and 3 and subsequently used to perform affinity purifications and quantitative mass spectrometry.

### Generation of clonal mutant L. tarentolae lines.

L. tarentolae promastigote mutants were generated using the adapted CRISPR-Cas9 protocol of Beneke et al. (33, 62, 63). To target the gene of interest, single guide RNA (sgRNA)-encoding sequences were designed using the Cas-OFFinder online tool (64). In general, the donor DNA for the repair of Cas9-induced double-strand breaks was amplified by PCR. The donor DNA comprised a selection marker cassette that was flanked by gene-specific 30-nucleotide homology arms at the 5′ and 3′ ends. Primer sequences for the generation of sgRNA templates and the amplification of targeting cassettes are listed in Table S1a and b. The donor DNA for the generation of chromosomally encoded *Lt*Erv*-*His_8_ was obtained by PCR with primers 149 and 167 and pPLOTv1 puro-mCherry-puro (62, 63) as a template. To introduce chromosomal *ERV* mutations in L. tarentolae and to avoid the skipping of desired mutations, we cloned plasmids pPLOT-His_8_-*ERV_recodon_*-His_8_-PURO and pPLOT-His_8_-*ERV_recodon_*-His_8_-BLAST as donor DNA templates. First, a recodonized sequence of *ERV* that was flanked by the 5′ regulatory element from the Crithidia fasciculata gene B for phosphoglycerate kinase, as well as MluI and SacI restriction sites, was synthesized and cloned into vector pUC57 (General Biosystems, Durham, USA). Degenerate codons were chosen to maintain the codon adaptation index value (65) of wild-type *ERV*. The synthetized fragment was excised from pUC57 using MluI and SacI and subcloned into pPLOTv1 puro-mCherry-puro or pPLOTv1 blast-mNeonGreen-blast (62, 63), yielding pPLOT-His_8_-*ERV_recodon_*-His_8_-PURO and pPLOT-His_8_-*ERV_recodon_*-His_8_-BLAST. The DNA sequence of the inserts was confirmed and is listed at the end of the supplemental material. The donor DNA for the generation of *ERV* mutants was amplified by PCR using plasmid pPLOT-*His_8_*-*ERV_recodon_*-*His_8_-PURO* or pPLOT-*His_8_*-*ERV_recodon_*-*His_8_-BLAST* as the template.

Transfections with the sgRNA-encoding DNA and donor DNA from unpurified PCR products were performed with 5 × 10^6^ promastigotes of a parental L. tarentolae line that transiently expressed Cas9 and T7 RNA polymerase from plasmid pTB007 (33). The Lonza Nucleofactor IIb program X-001 was used for electroporation. Cells were allowed to recover in 2 ml BHI medium without antibiotics for 16 h before selection on BHI agar plates with 20 μg/ml puromycin and/or 10 μg/ml blasticidin. Single colonies appeared 7 to 14 days after transfection and were subsequently transferred to liquid medium. The genome modifications were confirmed for clonal cell lines by analytical PCR using the primers in Table S1b and c as well as by Sanger sequencing (SEQ-IT GmbH & Co.KG) of PCR products with genomic DNA as a template.

### Generation of L. tarentolae lines with plasmid-encoded *Lt*Erv.

Primers for the cloning of *LTERV* constructs into vector pX-backbone are listed in Table S1d. To generate plasmid pX-backbone, a His_8_-encoding sequence followed by a XbaI and a HindIII restriction site was cloned into the BamHI and NotI restriction sites of plasmid pX using an annealing product of two complementary primers with matching sticky ends. *LTERV* and *LTERV^C63S^* were PCR-amplified using the corresponding pQE30 plasmids as templates (28) and were cloned into either the BamHI and HindIII restriction sites of plasmid pX-backbone, to obtain plasmids pX-*LTERV* and pX-*LTERV^C63S^*, or the XbaI and HindIII restriction sites of plasmid pX-backbone, to obtain plasmids pX-His_8_-*LTERV* and pX-His_8_-*LTERV^C63S^*. Transfections were carried out with 20 μg DNA in 10 μl sterile water using Nucleofactor IIb program U-033, and parasites were selected on BHI agar with 40 μg/ml G418 disulfate as described previously (33).

### *In situ* disulfide trapping experiments.

Wild-type parasites and promastigotes with plasmid-encoded upregulated His_8_-*Lt*Erv, *Lt*Erv^C63S^, or His_8_-*Lt*Erv^C63S^ were treated with either the thiol oxidizing agent diamide or the reducing agent DTT prior to thiol-blocking with either NEM or MMTS to stabilize potential protein-protein disulfide bonds *in situ*. All solutions were always freshly prepared. Different concentrations of NEM or MMTS were tested in the first set of experiments as indicated, whereas all subsequent experiments were carried out with 100 mM NEM. Parasites (5 × 10^7^ cells/sample) were harvested by centrifugation at 1,500 × *g* for 10 min at room temperature and were treated with either 2 mM diamide or 2 mM DTT in phosphate-buffered saline (PBS) for 1 to 120 min. Cells were subsequently centrifuged again and incubated with 100 mM NEM in PBS for 5 min at room temperature. Samples that were treated with PBS or 100 mM NEM in PBS served as controls. Afterward, the cells were centrifuged at 10,000 × *g* for 1 min at 4°C and resuspended and boiled in 5 × Laemmli buffer that contained 10% of the initial NEM or MMTS concentration and that was supplemented with or without 15% mercaptoethanol.

Three alternative alkylation treatments were tested for *in situ* trapping experiments with chromosomally encoded *Lt*Erv-His_8_ and *Lt*Erv^ΔKISS^-His_8_. Treatment I was performed with modifications as reported previously for HEK293 lines (66). Parasites (4 × 10^7^ cells) were harvested at 1,500 × *g* for 3 min, washed once in 500 μl ice-cold PBS supplemented with 100 mM NEM, and incubated for 30 min at room temperature. Cells were resuspended in 40 μl buffer containing 30 mM Tris, pH 8.1, 150 mM NaCl, and 100 mM NEM and subsequently lysed by adding 10 μl of 8% SDS. For treatment II, 4 × 10^7^ parasites were harvested at 1,500 × *g* for 3 min and treated with warm BHI medium that was supplemented with 20 mM diamide. Cells were incubated for 10 min at 27°C and further processed as described for treatment III. For treatment III, 4 × 10^7^ parasites were harvested and immediately resuspended in 500 μl ice-cold 10% TCA. Cells were lysed by freeze-thawing in liquid nitrogen and incubated for 20 min on ice. Proteins were precipitated by centrifugation at 30,000 × *g* for 5 min, washed twice with 2 ml ice-cold acetone, and dried for 5 min at room temperature. The protein pellet was subsequently resuspended in 50 μl buffer containing 100 mM Tris-HCl, pH 7.0, 2% SDS, and 100 mM NEM and incubated for 30 min at 37°C. All samples were analyzed by nonreducing and reducing SDS-PAGE followed by Western blotting using either a mouse anti-6×His (Thermo Scientific) or a rabbit anti-*Lt*Erv primary antibody (2).

### Protein purification.

His-tagged bait proteins from SILAC experiments were purified by affinity chromatography based on a modified protocol for HEK293 lines (66). Labeled and nonlabeled parasites (∼35 × 10^7^ cells) were harvested by centrifugation at 1,500 × *g* for 5 min at 4°C. To prevent thiol-disulfide exchange reactions of putative mixed intermediates, intact parasites were subsequently resuspended in 3 ml ice-cold PBS containing 100 mM NEM, centrifuged at 1,500 × *g* for 5 min at 4°C, resuspended again in 2.5 ml PBS containing 100 mM NEM, and incubated for 10 min at 4°C. The cell suspensions from labeled and nonlabeled cultures were mixed 1:1, centrifuged at 1,500 × *g* for 5 min at 4°C, and resuspended in 4 ml sample buffer containing 30 mM Tris, pH 8.1, 150 mM NaCl, 100 mM NEM, and 10 μM EDTA. Cell lysis and protein denaturation were carried out in 200-μl fractions by adding 50 μl 8% SDS and sample heating for 20 min at 96°C. Afterward, 750 μl of sample buffer containing 2.5% Triton X-100 was added and incubated for 1 h at 4°C. The lysates were subsequently cleared by centrifugation at 30,000 × *g* for 1 h at 4°C and incubated with 100 μl equilibrated Ni-NTA agarose at 4°C overnight. Beads were first washed thrice with 1 ml sample buffer that was supplemented with 1.6% SDS and 1.9% Triton X-100 and then washed thrice with 1 ml PBS. Proteins were eluted with 100 μl PBS that was supplemented with 400 mM imidazole and heated at 95°C for 10 min.

### Mass spectrometry and data processing.

Protein samples were loaded on SDS-PAGE gels and allowed to migrate approximately 8 mm into the separating gel. Protein bands were cut out, followed by tryptic in-gel protein digestion and peptide desalting as described in Veyel et al. (67). Peptides were resuspended in a solution of 2% acetonitrile and 1% formic acid just before the liquid chromatography-tandem mass spectrometry (LC-MS/MS) run. The LC-MS/MS system (Eksigent nanoLC 425 coupled to a TripleTOF 6600; ABSciex) was operated as described in Hammel et al. (68). Quantitative analysis of mass spectrometry measurements was performed using MaxQuant 1.6.0.1 (69). Peptide spectrum matching was performed by constructing a spectral library based on release 42 of the TriTrypDB L. tarentolae Parrot Tar II proteome (52). The peptide library was generated considering methionine oxidation, acetylation of protein N termini, and cysteine alkylation as variable modifications. To identify proteins of cells that were grown in heavy medium, the search space was augmented by sequences containing [^13^C]_6_-l-arginine and [^13^C]_6_-l-lysine. False-discovery rate (FDR) thresholds for peptide spectrum matching and protein identification were set to 0.01. Besides quantification based on labeled peptides, proteins were also quantified using the label-free quantification (LFQ) algorithm (70). The mass spectrometric proteomic data are available to the scientific community via the ProteomeXchange Consortium partner repository PRIDE (71) with the data set identifier PXD027121. A protein was not considered a putative interaction partner of *Lt*Erv if it contained peptides that mapped to a putative contaminant or a decoy protein or if it was quantified in fewer than 3 replicates. After log-transforming the light-to-heavy (L/H) ratios of the remaining set of proteins, we computed the mean and the 95% confidence interval using all replicates. Subsequently, we fitted a gaussian with mean, G_m_, and standard deviation, G_s_, to the distribution of the means. As most of the proteins do not interact specifically, the distribution was observed to center around G_m_ of ∼0. A protein was considered an interaction partner if the computed 95% confidence interval exceeded Tukey’s outlier criterion given by G_m_ ± (1.96 · G_s_). The *Lt*Erv-His_8_ versus *Lt*Erv^ΔKISS^-His_8_ ratio was computed based on a set of peptides shared by both isoforms.

### Bioinformatic sequence analyses.

Amino acid sequences for Erv homologues from kinetoplastida were retrieved from TriTrypDB (52) and used to generate a multiple-sequence alignment and phylogenetic tree with Clustal Omega (EMBL-EBI) in order to calculate residue conservation scores with ConSurf (72).

### Data availability.

All relevant data are included in the article or the supplemental material and are available from the authors upon request. The mass spectrometric proteomic data are available to the scientific community via the ProteomeXchange Consortium partner repository PRIDE (71) with the data set identifier PXD027121.
