# Supplementary material for: In Vivo Structure-Function Analysis and Redox Interactomes of Leishmania tarentolae Erv
Source: Microbiol Spectr. 2021 Sep 29;9(2):e00809-21. doi: 10.1128/Spectrum.00809-21 (PMC8557897; doi:10.1128/Spectrum.00809-21)
Supplement: SUPPLEMENTAL FILE 1 — Supplemental material. Download SPECTRUM00809-21_Supp_1_seq1.pdf, PDF file, 2.1 MB [file spectrum00809-21_supp_1_seq1.pdf]

Supplementary Material and Methods

In vivo structure-function analysis and redox interactomes of *Leishmania tarentolae* Erv

Gino L. Turra[1,2], Linda Liedgens[1,2], Frederik Sommer[3], Luzia Schneider[2], David Zimmer[4], Jordi Vilurbina Perez[2], Sasa Koncarevic[5], Michael Schroda[3], Timo Mühlhaus[4], and Marcel Deponte[1,2]*

[1]Department of Parasitology, Ruprecht-Karls University, D-69120 Heidelberg, Germany

[2]Faculty of Chemistry, TU Kaiserslautern, D-67663 Kaiserslautern, Germany

[3]Molecular Biotechnology & Systems Biology, TU Kaiserslautern, D-67663 Kaiserslautern, Germany

[4]Computational Systems Biology, TU Kaiserslautern, D-67663 Kaiserslautern, Germany

[5]Proteome Sciences R&D GmbH & Co. KG, 60438 Frankfurt am Main, Germany

*Correspondence to: Marcel Deponte

Faculty of Chemistry, TU Kaiserslautern, D-67663 Kaiserslautern, Germany

Phone: +49 631 205 3421

Email: deponte@chemie.uni-kl.de

Supplementary Figure 1

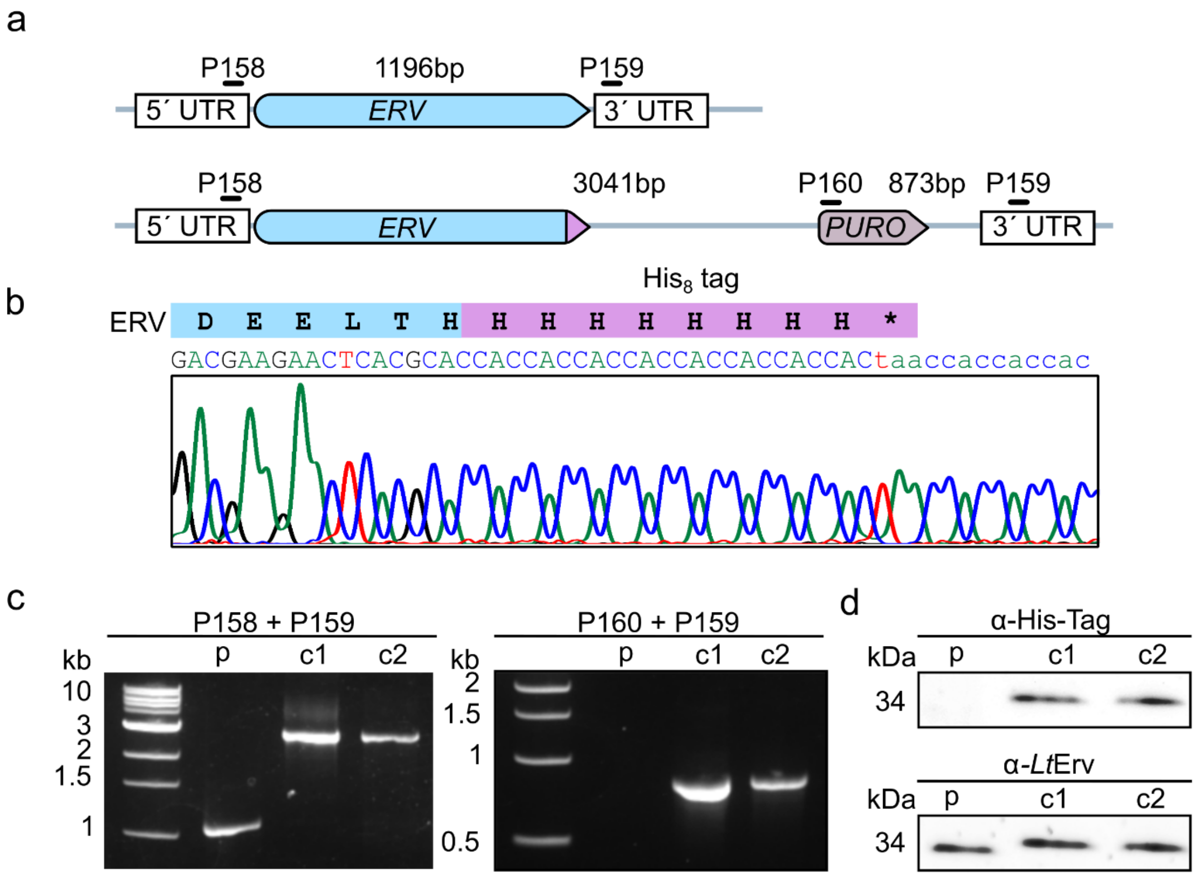

Fig. S1. *Generation and validation of L. tarentolae strains with chromosomally encoded LtErv-His$_8$.* (**a**) Schematic representation of the loci for wild-type *LTERV* (top) and *LTERV-HIS$_8$* with an antibiotic resistance cassette against puromycin (bottom). Only one locus is shown for diploid parasites. Primer binding sites and expected product sizes from analytical PCRs are indicated. (**b**) Section of a Sanger sequencing chromatogram from a representative PCR amplicon of a homozygous clone encoding *Lt*Erv-His$_8$. (**c**) Genotyping of the parental strain (p) and two clonal strains (c1 and c2) that were obtained after CRISPR-Cas9 editing and selection with puromycin. Analytical PCRs with the indicated primers from panel a confirmed the homologous recombination and the integration of the resistance cassette. (**d**) Western blot analysis of clones c1 and c2 with *Lt*Erv-His$_8$ with a calculated molecular mass of 35.7 kDa. The parental strain (p) with untagged *Lt*Erv served as control. Alternative decorations with antibodies against the His$_8$-tag (top) or against *Lt*Erv (bottom) confirmed the specificity of the signal.

Supplementary Figure 2

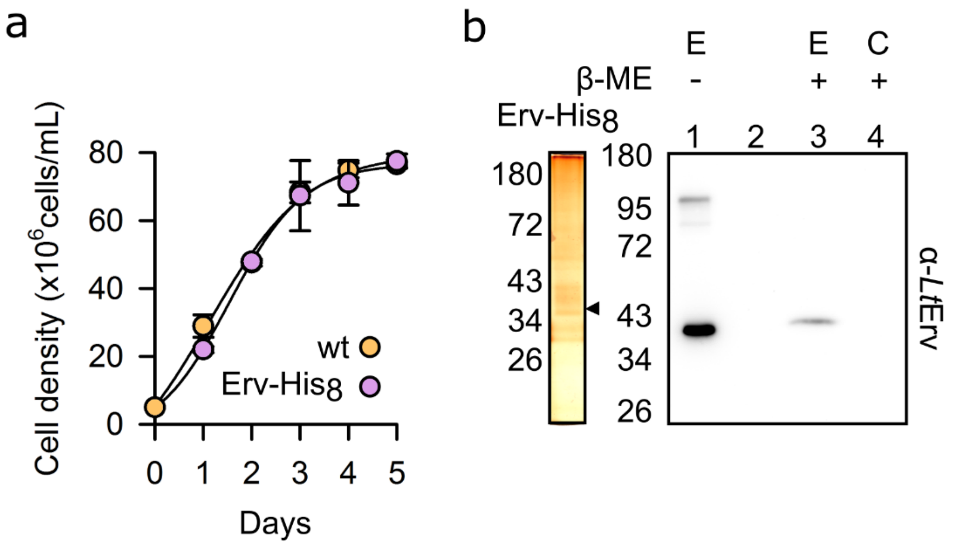

Fig. S2. *Growth phenotype and protein purification from L. tarentolae promastigotes with LtErv-His*$_8$*.* (**a**) Growth curve analysis in BHI liquid medium of *L. tarentolae* strains with wild-type *Lt*Erv (wt) or tagged *Lt*Erv-His$_8$. Data points represent the mean ± standard deviation from two independent biological replicates. (**b**) Purification of *Lt*Erv-His$_8$ from a cell lysate by affinity chromatography with Ni-NTA agarose. The protein content of the eluate was analyzed by SDS-PAGE and silver staining. *Lt*Erv-His$_8$ was detected by western blot analysis following SDS-PAGE with or without β-mercaptoethanol (β-ME). Parallel purifications of *Lt*Erv-His$_8$ and *Lt*Erv from mutant and wild-type cells confirmed the specific enrichment of the tagged protein in the eluate (E) in contrast to the negative control (C), respectively.

Supplementary Figure 3

a

Components	Conditions 1	2	3	4	5
M199	+	+	+	+	+
FBS	-	+	-	-	-
dFBS	+	-	-	-	+
Hypoxanthine	+	+	+	-	-
6-Biopterin	+	+	+	-	-
Biotin	+	+	+	-	-
Folic Acid	+	+	+	-	+
Adenosine	+	+	+	-	+
L-Glutamine	+	+	+	-	+
L-Proline	-	-	-	-	+
HEPES	+	+	+	+	+
Hemin	+	+	+	+	+

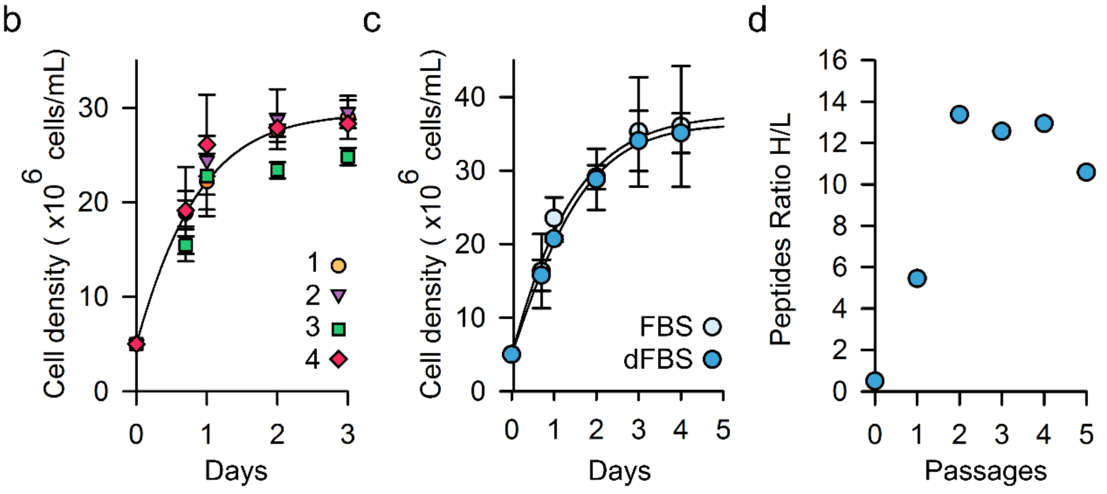

Fig. S3. *Identification and validation of a suitable medium for SILAC experiments with L. tarentolae.* (**a**) Overview of the variations of supplemented M199 with or without dialyzed or normal fetal bovine serum (dFBS and FBS, respectively). (**b**) Growth curve analysis of wild-type promastigotes for conditions 1-4. (**c**) Growth curve analysis of wild-type promastigotes for condition 5 with FBS or dFBS. (**d**) Calculated incorporation efficiency based on the heavy/light (H/L) ratios of all identified peptides. For each passage, cultures were diluted 1:20 every 72 h. Whole-cell lysates were prepared from the remaining undiluted cell culture and analyzed by mass spectrometry. Each data point in panels b and c represents the mean ± standard deviation from two independent biological replicates.

Supplementary Figure 4

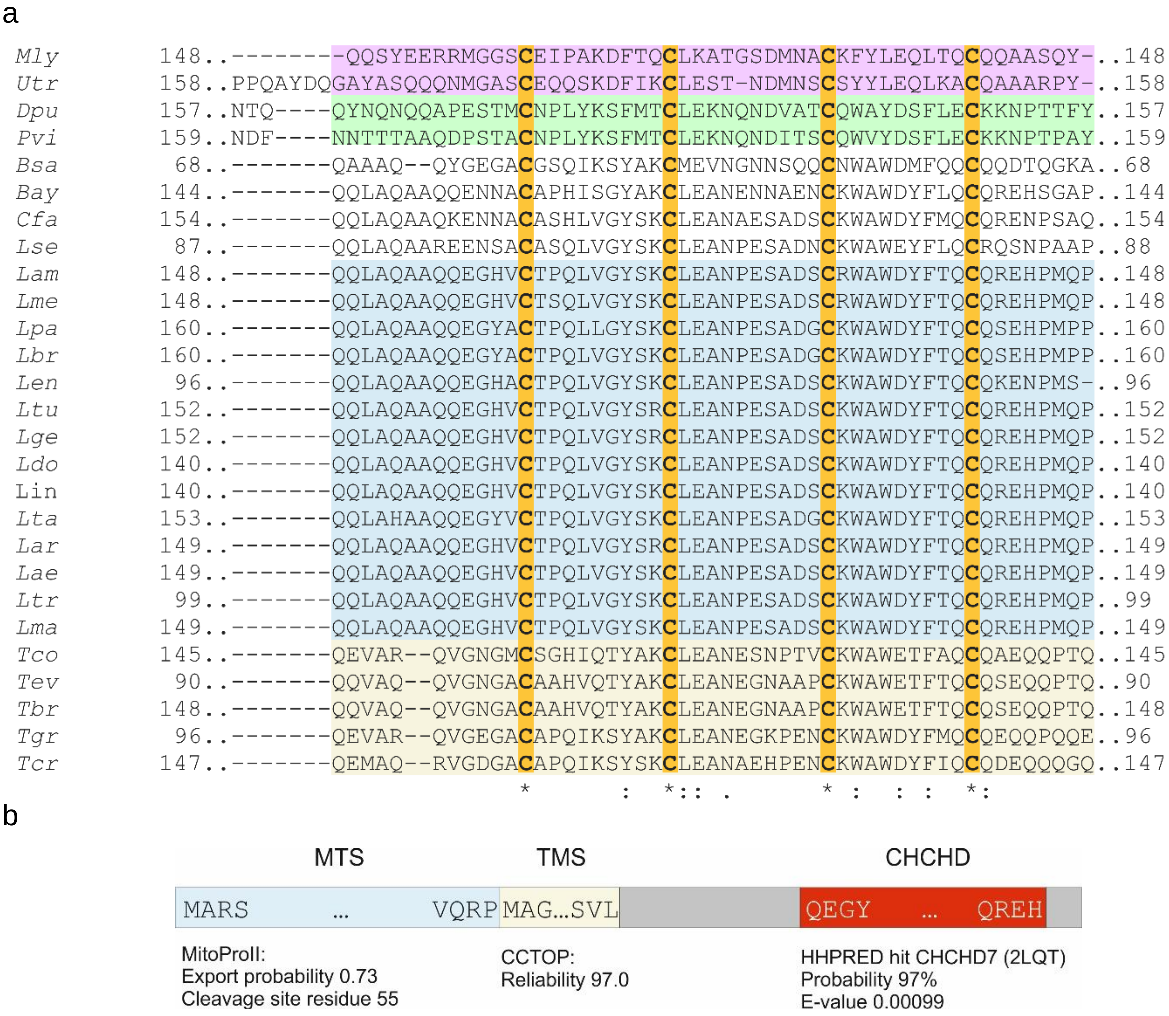

Fig. S4. *Multiple sequence alignment and predicted properties of candidate interaction partner LtaP04.0660 (GET85674.1) from Fig. 2.* (**a**) BLAST searches revealed conserved homologues in diverse kinetoplastida (shaded in white, blue, and yellow) and also in a few opisthokonts (shaded in purple) as well as very few amoebozoa (shaded in green). The twin Cx_9C motif is highlighted. (**b**) Predicted protein properties. MTS, mitochondrial targeting sequence; TMS, transmembrane segment; CHCHD, coiled-coil-helix-coiled-coil-helix domain. *Mly, Microbotryum lychnidis-dioicae; Utri, Ustilago trichophora; Dpu, Dictyostelium purpureum; Pvi Polysphondylium violaceum; Bsa, Bodo saltans; Bay, Blechomonas ayalai; Cfa, Crithidia fasciculate; Lse, Leptomonas seymouri; Lam, L. amazonensis; Lme, L. mexicana; ; Lpa, L. panamensis; Lbr, L. braziliensis; Len, L. enrietti; Ltu, L. turanica; Lge L. gerbilli; Ldo, L. donovani; Lin L. infantum; Lta, L. tarentolae; Lar, L. arabica; Lae, Leishmania aethiopica; Ltr, L. tropica; Lma, L. major; Tco, T. congolense; Tev, T. evansi; Tbr, Trypanosoma brucei; Tgr, T. grayi; Tcru, T. cruzi*

Supplementary Figure 5

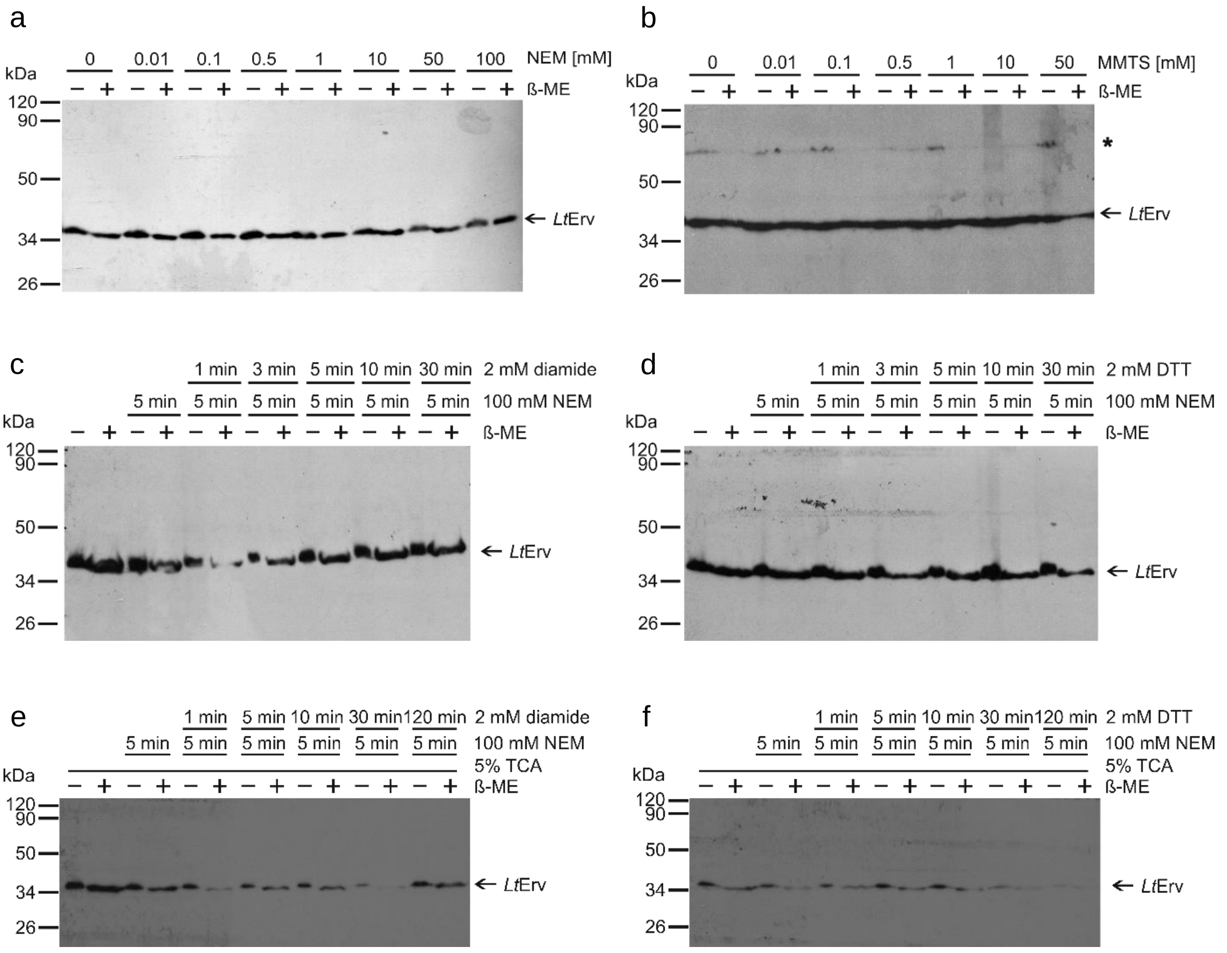

Fig. S5. *Chemical redox trapping experiments for LtErv from wild-type L. tarentolae promastigotes.* SDS-PAGE and western blot analyses of Laemmli samples with 5×10^7 parasites per lane. Samples were analyzed under reducing and non-reducing conditions with or without β-ME. (**a**) and (**b**) Parasites were treated for 5 min with the indicated concentrations of the alkylating agent NEM or MMTS to block thiols and to inhibit thiol-disulfide exchange reactions. The asterisk labels a band that might indicate dimeric *Lt*Erv and that was sometimes detected under non-reducing conditions regardless of the absence or presence of alkylating agents. (**c**) and (**d**) Parasites were treated with diamide or DTT for 1-30 min to promote or prevent the formation of disulfide bonds before the addition of NEM. The calculated molecular mass of monomeric *Lt*Erv is 34.8 kDa. *Lt*Erv was detected using a specific antibody (1). (**e**) and (**f**) Parasites were treated for the indicated times points with diamide or DTT before the addition of NEM and subsequent cell lysis and protein precipitation with 5% TCA.

Supplementary Figure 6

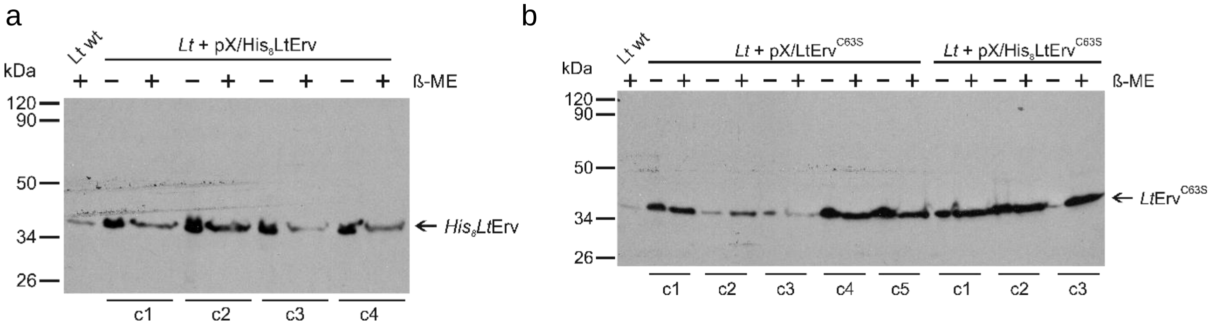

Fig. S6. *Redox state of LtErv in strains with upregulated His_8-LtErv, $LtErv^{C63S}$* or *His_8-$LtErv^{C63S}$.* Parasites with chromosomally encoded *Lt*Erv were transfected with plasmid pX encoding (**a**) His_8-*Lt*Erv or (**b**) tagged or untagged *Lt*Erv^{C63S}. G418-resistant parasites were cultured and treated for 5 min with 100 mM NEM before cell lysis. SDS-PAGE and western blot analyses were performed with Laemmli samples containing 5×10^7 *L. tarentolae* promastigotes per lane. Samples were analyzed under reducing and non-reducing conditions with or without β-ME. The *Lt*Erv-content was compared with the wild-type strain using an antibody against *Lt*Erv.

Supplementary Figure 7

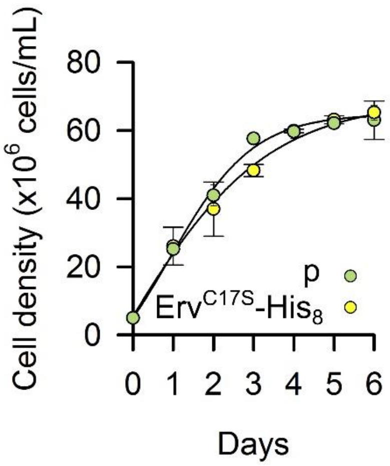

Fig. S7. *Growth phenotype of L. tarentolae strains with His*$_8$*-LtErv*^{C17S} *or LtErv*$^{\Delta KISS}$*-His*$_8$*.* Growth curve analysis in BHI liquid medium of *L. tarentolae* strains with His$_8$-*Lt*Erv^{C17S}. The parental strain (p) served as a control. Data points represent the mean ± standard deviation from two independent biological replicates.

Supplementary Figure 8

a

```
Emo   ---MIRDQCHAYCKCSYCAQQP..207..CENAPHVCGHCLDVQVQAR..471
Len   ---MIRDQCHAYCQCAYCAQKP..207..CENQPHVCGHCLDVDVQAR..471
Lbr   ---MIRDQSHAYCQCSYCTQQP..207..CEHQPHVCNHCLDVNVQAR..467
Lpa   ---MIRDQSHAYCQCSYCTQQP..207..CEHQPHVCNHCLDVNVQAR..465
Lta   ---MIRDQCHAYCRCSYCAQEP..208..CENQPHVCGHCLDVDVQAR..468
Lam   ---MIRDQCHAYCRCSYCAQQP..207..CENQPHVCGHCLDVNVQAR..467
Lme   ---MIRDQCHAYCRCSYCAQQP..207..CENQPHVCGHCLDVNVQAR..467
Ldo   ---MIRDQCHAYCRCSYCAQQP..206..CENQPHVCGHCLDVNVQAR..466
Lin   ---MIRDQCHAYCRCSYCAQQP..206..CENQPHVCGHCLDVNVQAR..466
Ltr   ---MIRDQCHAYCRCSYCAQQP..207..CENQPHVCGHCLDVNVQAR..467
Lae   ---MIRDQCHAYCRCSYCAQQP..207..CENQPHVCGHCLDVNVQAR..470
Lge   ---MIRDQCHAYCRCSYCAQQP..208..CENQPHVCGHCLDVNVQAR..470
Lma   ---MIRDQCHAYCRCSYCAQQP..207..CENQPHVCGHCLDVNVQAR..470
Lar   ---MIRDQCHAYCRCSYCAQQP..207..CENQPHVCGHCLDVNVQAR..470
Ltu   ---MIRDQCHAYCRCSYCAQQP..207..CENQPHVCGHCLDVNVQAR..470
Cfa   ---MIRDQCHAYCQCSYCAQEP..213..CANQPHLCGHCMDVDVQAR..474
Lpy   ---MIRDQCHAYCQCSFCSQEP..215..CENQPHVCGHCLDVNVQAR..426
Lse   ---MIRDQCHAFCQCSYCTQEP..214..CENQPHVCGHCLDFNVQAR..477
Tcr   ---MIRDQCKAYCQCS-CKQSG..194..CTEQPHACNHCFSINAQGK..422
Tth   ---MIRDQCKAYCQCSYCANNK..198..CPMQPHMCGHCFAVNAQGK..445
Tra   ---MIRGQCKTYCQCNYCKQPR..190..CPLQPHTCGHCFAVNVQGK..441
Tgr   ---MIRDQCKTFCRCSYCEQQK..195..CPDQPHMCNHCFAVNAQGK..415
Tvi   ---MLHTQCKAFCCCSYCKGRR..191..CPEDEHLCGHCFAVNAQGK..385
Tev   ---MLREQCKDFCRCSYCEKKR..192..CPEDEHLCGHCFAVNAQGK..420
Tbr   ---MLREQCKDFCRCSYCEKKR..192..CPEQPHLCGHCFAVNAEGK..420
Tco   MGEVLRQQCMDFCCCTYCRRKR..193..HKCQVPPAHVLRDSL----..411
         ::: *.  :* *  *           .    * ..*
```

b

```
Pco   1351..SVWPLLCRCAILPLYGSKSVYSRD..1838
Cfa   2108..ASWTRLCTCRLLPIYGSKPAYPRE..2575
Lse   1982..ASWTRLCTCRLLPLYGSKPAYPRE..2446
Bay   72  ..ASWTNLCTCKLLPLYGAQPVYSRD..541
Emo   2121..ASWTRLCTCRLLPLYGSKPAYPPE..2595
Len   2045..ASWTRLCTCRLLPLYGSKPAYPRE..2519
Lbr   2000..ASWTHLCTCRLLPLYGSKPAYPRE..2473
Lpa   1909..ASWTRLCTCRLLPLYGSKPAYPRE..2377
Lta   2094..ASWTRLCTCRLLPLYGSKPAYPRE..2568
Lam   1808..ASWTRLCTCRLLPLYGSKPAYPRE..2282
Lme   1809..ASWTRLCTCRLLPLYGSKPAYPRE..2283
Lma   2062..ASWTRLCTCRLLPLYGSKPAYPRE..2541
Lar   2053..ASWTRLCTCRLLPLYGSKPAYPRE..2532
Ltu   2062..ASWTRLCTCRLLPLYGSKPAYPRE..2541
Lge   2061..ASWTRLCTCRLLPLYGSKPAYPRE..2540
Lin   1839..ASWTRLCTCRLLPLYGSKPAYPRE..2317
Ldo   2094..ASWTRLCTCRLLPLYGSKPAYPRE..2572
Ltr   2099..ASWTRLCTCRLLPLYGSKPAYPRE..2573
Lae   2094..ASWTRLCTCRLLPLYGSKPAYPRE..2568
Tco   1102..SPWSALCYCRILPLFGVKAVYPRD..1549
Tev   1075..SHWSALCHCRILPLFGAKPAYPRD..1519
Tbr   1075..SHWSALCYCRILPLFGAKPAYPRD..1519
Tvi   1021..SLWASLCLCKALPLYGMRSAYPRD..1476
Tth   1071..SPWVSLCHCKILPLYGTKSAYPRD..1518
Tgr   1139..SPWAALCHCKILPLYGMKSAYPRD..1519
Tcr   1139..SPWATLCHCKILPIYGTKSPYQRD..1586
            : *  ** *  **::* :  *  :..
```

Fig. S8. *Multiple sequence alignments for candidate interaction partners of LtErv.* (**a**) Multiple sequence alignment for UF1 (LtaP32.0380) from Fig. 6. (**b**) Multiple sequence alignment for UF2 (LtaP07.0980) from Fig. 6. Sequences from *Leishmania* and *Trypanosoma* species are shaded in blue and yellow, respectively.

Conserved cysteine motifs are highlighted. *Bay, Blechomonas ayalai; Cfa, Crithidia fasciculate; Emo, Endotrypanum monterogeii; Lae, Leishmania aethiopica;Lam, L. amazonensis;Lar, L. arabica; Lbr, L. braziliensis; Ldo, L. donovani; Len, L. enriettii; Lge L. gerbilli; Lin L. infantum; Lma, L. major; Lme, L. mexicana; Lpa, L. panamensis; Lta, L. tarentolae; Ltr, L. tropica; Ltu, L. turanica; Lpy Leptomonas pyrrhocoris; Lse, Leptomonas seymouri; Pco, Paratrypanosome. confusum; Tbr, Trypanosoma brucei; Tco, T. congolense; Tcru, T. cruzi; Tev, T. evansi; Tgr, T. grayi; Tth, T. theileri; Tvi, T. vivax*.

Supplementary Table 1. List of primers.

**a) Universal sgRNA antisense primer**	
**Primer name**	**Sequence**
p/GOO	5'-AAAAGCACCGACTCGGTGCCACTTTTTCAAGTTGATAACGGACTAGCCTTATTTTAACTTGC TATTTCTAGCTCTAAAAC-3'

b) Primers used for the generation of CRISPR-Cas9 mutants of *LTERV*. List of primers used to generate targeting cassettes and sgRNA templates for chromosomally encoded His_8-tagged and mutated version of *Lt*Erv. Mutated nucleotides are bold and italicized, gRNA spacer sequences are underlined. See materials and methods for details.

**Primer name**	**Number**	**Sequence**
p1/ERV1KO/Fw	148	5'-GGTGCCTATCCTCATAGCCAGGCTCCACTTgtataatgcagacctgctgc-3'
p5/ERV1KO/Rv	149	5'-TCAGTGCCTCTTGCGATATCTACACTGCCGccaatttgagagacctgtgc-3'
p4/ERV1Ctag/Fw	153	5'-TACTGCCCAGAGGACGAAGAACTCACGCACggttctggtagtggttccgg-3'
p/ERV1sgRNA5'/01	150	5'-gaaattaatacgactcactataggTCCCTCGCTCGCTCGCTCGCgttttagagctagaaatag-3'c
p/ERV1sgRNA3'/02	151	5'-gaaattaatacgactcactataggCAGCCATGATGGCTCGCAGAgttttagagctagaaatagc-3'
p/ERV/5'UTR/Fw	158	5'-TCACCAATCACCGACCCTTTC-3'
p/ERV/3'UTR/Rv	159	5'-GCGTATCTCTTACACGTATACC-3'
p4/Plot/ERVCHis8/Fw	167	5'-TACTGCCCAGAGGACGAAGAACTCACGCACCACCACCACCACCACCACCAC CACtaaccaccaccaccactgag-3'
p4/ERVY303P/Fw	187	5'-GTTCTCAAACGTCTCAAGCGTTGTCAAGTG***cca***TGCCCAGAGGACGAAG-3'
p4/ERVY303D/Fw	188	5'-GTTCTCAAACGTCTCAAGCGTTGTCAAGTG***gat***TGCCCAGAAGACGAAG-3'
P4/ERVCPC/Fw	189	5'-GATGCCGTTCTCAAACGTCTCAAGCGTTGT***cca***TGCCCAGAAGACGAAG-3'
p/ERVsgRNAORF/02	190	5'-gaaattaatacgactcactataggTTGTCAAGTGTACTGCCCAGgttttagagctagaaatagc-3'
p/ERVNterSeq/Fw	191	5'-CCACCGCGGAGGTGTACCAG-3'
p4/ERVY303P/r/Fw	203	5'-GTTCTCAAACGTCT***t***AAGCGTTGTCAAGTGccatgtcctgaagatgagg-3'
p4/ERVCPC/r/Fw	204	5'-GATGCCGTTCTCAAACGTCT***t***AAGCGTTGTccatgtcctgaagatgagg-3'
p4/ERVY303D/r/Fw	205	5'-TTCTCAAACGTCT***t***AAGCGTTGTCAAGTGgattgtcctgaagatgagg-3'
P4/ERVR299D/r/Fw	206	5'-GACATCGATGCCGTTCTCAAACGTCT***t***AAGgattgccaggtatactgtc-3'
p/ERVsgRNANORF/03	207	5'-gaaattaatacgactcactataggTGGTGAGTGCCCCACCCCGCgttttagagctagaaatagc-3'
p2/ERVC17S/rec/Rv	208	5'-CCCTGACACGCCCAGCTCCAGCGGGGTGGGtgattcgcccgggattgtg-3'

p/ERVrecoseq/Fw	209	5'-catatgcaccaccaccaccacc-3'
p/ERVsgRNAKISS/01	210	5'-gaaattaatacgactcactataggGGCGTATCCTCCATCTTGTTgttttagagctagaaatagc-3'
P4/LTERVKISS/Fw	211	5'-GTGGTGCTGCGTCGGTGGCACCCTGGGTACccaaccctgccgcgcg-3'
P5/LTERVKISS/Rv	212	5'-TTCGATGCCGCTAGTTGTTCCTCGATCGTTccaatttgagagacctgtgc-3'
p5/LTERV1KO/Rv/02	214	5'-TGCCTGCCCTAGTGCGTGAGTTCTTCGTCCTccaatttgagagacctgtgc-3'

c) General genotyping primers.

**Primer name**	**Number**	**Sequence**
p/Puromycin/Fw	160	5'-ATGACTGAATACAAGCCAACGG-3'
p/Puromycin/Rw	139	5'-TCAATGTGTCGATCTGGGTCAAC-3'
p/Blasticidin/Rw	140	5'-CCGTTGCTCTTTCAATGAGGGTG-3'

d) Primers used for the generation of pX-*LTERV* constructs. The coding sequence for the His_8-tag is italicized and restriction sites are highlighted. See materials and methods for details.

**Primer name**	**Sequence**
p/pX/backbone/s	5'-GATCC**ATG***CATCATCACCATCATCACCATCAC*TCTAGAAAGCTTGC-3'
p/pX/backbone/as	5'-GGCCGCAAGCTTTCTAGA*GTGATGGTGATGATGGTGATGATG*CATG-3'
p/LTERV/BamHI/s	5'- GATCGGATCC**ATG**TCGGACGACGACGTACACG-3'
p/pX/LTERV/HindIII/as	5'-GATCAAGCTTTTAGAGCTTGAGTTCTTC-3'
p/pX/LTERV/XbaI/s	5'-GATCTCTAGATCGGACGACGACGTACACG-3'

Insert sequence of pPLOT-His$_8$-*ERVrecodon*-His$_8$-PURO and pPLOT-His$_8$-*ERVrecodon*-His$_8$-BLAST constructs

*acgcgt*tcgagaccgacaagaccagaatagcgcttatgtgtgtgtgtttgtgtgtgggtgtgtgtgcccat
gtgtgacaaagaggccagtgctttctctaaagcaagagcgcgaggttgctggctaaggactctaagtccct
cacagtgatggcgtgttgcattttctaaaccgtcaatccaacacgtcggtagcatatcggttttgtaggct
cttctggataccttccccagtaagaccgccactgcttcgtgactgaagcactgtaaatatcactgaaaccg
tcgcgttgctttgtctaattatcaccgcctttgccagctatcgcctcaggctgcgctctttgactaatcca
ctcaccacttctctgccttcttcctccggttgttgttgtgtcctgcgtgtacatggcgcgtgtccttttcg
agcaaacagctgtcttttctttcacgataacacactcatattaaacgcgagtattactcatcagtcaacgt
cacattccgctctgtccacttcgaccttacacctctacttgtccaactatcttccacttgtcaagc*cat**AT*
GCACCACCACCACCACCACCACCACTCGGATGACGGCGTGCAAGAGAGGCTGACCACAATCCCGGGCGAAT
GCCCTACTCCTCTCGAACTGGGCGTGTCCGGCTGGAACATCCTGCACAGCTCGGCGGCCGTGTACCCCTAC
AAGCCGAGCAGCGTGCAGCAGACCGCCATGCGCAACTTCATCGAGAGCTGGGCGTACGTGTACGCCTGCTC
GTGGTGCGCGTACCACATGCGCGAGTACGTGCGCGCCCACCCGCCCGACGTCCGCGATAAGCTGACGGTGA
GCCGCTACGTCTGCGAGATGCACAACAACGTGAACGAGCGCCTGGGCAAGGAGCTGTTTGACTGCACGCCG
AGCGTGGTCCTGCGCCGCTGGCACCCGGGCTACCCCAACAAGATGGAGGACACGCCGACCATTGAGGAGCA
GCTCGCAGCTTCGAACCGCGAGAAGTCCGCCGCAAAGGACAGCGCACACCACGAGCAGGAGCACGAGCGCC
GCTTCAGCGGTTTTGGCCTGCACGCACGCGAGGCAGCTTCCAACAAGGTGGTGGACCGTCGCGACGCGAGC
GAGGCCGCGCCACGCAAGGTGGACGGCGGCCTGTGGATCTGGCGCATTTTCCGCCGCCCAGTGCTGGGTAC
GCCGGCAGCTGAGAGCAACGCGAGCCCGGGTATGAAGAGCAGCAGCCCGCCGCCGACGGCCCAGCACGAGA
AGAACATCATTAGCGGTACCCGCGAGACGCCGCCGGCGGGTGCAGTGACCGCCGCGGCAAGCAACGTCAGC
GCTGCCCACTCCCGCCACAGCTGGGGCGCCACGAGCACCGCCGAGGTGTACCAGAGCGCAGCACTGCCAAC
CCTGCCGCGCGGTAAAGCGGCCGACGATGAAACGGATATCGACGCAGTGTTAAAGCGCTTAAAACGGTGCC
AGGTATACTGTCCTGAAGATGAGGAGCTGACACACCACCACCACCACCACCACCAC**TGA***ggatcc*ccacca
ccaccactgagaattctgtattacgccgttttaa*gagctc*

References

1. Eckers E, Cyrklaff M, Simpson L, Deponte M. 2012. Mitochondrial protein import pathways are functionally conserved among eukaryotes despite compositional diversity of the import machineries. Biol Chem 393:513-24.
